# Fatty Acid Metabolism and Hypoxia in the Tumor Microenvironment: Metabolic Adaptation and Clinical Potential in Colorectal Cancer

**DOI:** 10.3390/biomedicines14081712

**Published:** 2026-07-30

**Authors:** Junqi Zhang, Sian Xie, Yongjun Wang

**Affiliations:** Department of Gastroenterology, Beijing Friendship Hospital, Capital Medical University, State Key Laboratory of Digestive Health, National Clinical Research Center for Digestive Diseases, Beijing Key Laboratory for Precancerous Lesion of Digestive Diseases, No. 95, Yong An Road, Xi Cheng District, Beijing 100050, China; zhangjunqi@mail.ccmu.edu.cn

**Keywords:** colorectal cancer, fatty acid metabolism, hypoxia, hypoxia-inducible factor, fatty acid oxidation, lipid droplets

## Abstract

Colorectal cancer (CRC) is a major cause of cancer morbidity and mortality worldwide, and metabolic reprogramming is increasingly recognized as an important feature of its progression. Among these changes, fatty acid metabolism (FAM) has drawn growing attention because it supports energy supply, membrane synthesis, redox balance, and stress adaptation in tumor cells. CRC cells can increase fatty acid uptake, activate *de novo* synthesis, adjust fatty acid oxidation (FAO), and alter lipid droplet (LD) dynamics according to metabolic demand. These processes are strongly influenced by the hypoxic tumor microenvironment. Under hypoxic conditions, signaling pathways centered on hypoxia-inducible factors (HIFs) reshape lipid uptake, synthesis, oxidation, and storage, allowing CRC cells to maintain survival and adapt to limited oxygen and nutrient availability. Increasing evidence suggests that this metabolic shift is closely linked to invasion, metastasis, stem-like behavior, and resistance to therapy. In this review, we provide an integrated overview of the hypoxia–FAM axis in CRC. We first summarize the major steps of FAM reprogramming, then highlight how hypoxia reshapes these processes through HIF-dependent and related pathways. We also discuss FAM crosstalk with stromal and immune cells, experimental models, and metabolic heterogeneity between primary CRC and liver metastases. Finally, we discuss therapeutic strategies targeting FAM and hypoxia-associated signaling in CRC.

## 1. Introduction

Colorectal cancer (CRC) is one of the most common malignant tumors of the digestive system in the world. In 2024, CRC ranked third in terms of new cancer cases and second in cancer-related deaths globally, posing a serious threat to human health [1,2]. Despite advancements in early screening, endoscopic techniques, and comprehensive treatment strategies, improvements in CRC prognosis remain limited. Notably, the incidence of early-onset CRC has increased worldwide, accompanied by a substantial rise in the number of related deaths [3,4]. CRC development is linked to a number of factors, including genetic predisposition, environmental effects, and abnormal reprogramming of cellular metabolism, all of which play important roles in CRC initiation and progression [5,6,7].

Fatty acid metabolism (FAM), a critical component of tumor metabolic reprogramming, has attracted increasing attention in CRC, which is closely associated with obesity [8,9]. Fatty acids (FAs) are important metabolic substrates and constituents of cellular lipids, with roles in energy production and membrane formation [10,11]. Several enzymes involved in FA uptake, *de novo* synthesis, fatty acid oxidation (FAO), and FA storage are abnormally regulated in cancers, resulting in FAM reprogramming. Tumor cells can absorb exogenous free FAs or enhance endogenous lipogenesis to meet the high energetic demands of tumor metabolism and sustain malignant behavior [12,13,14,15].

The hypoxic microenvironment is a characteristic of solid tumors, and adaptive alterations of tumor cells under hypoxic conditions are key drivers of metabolic reprogramming. The hypoxic microenvironment can activate transcriptional regulatory pathways such as the hypoxia-inducible factor (HIF) pathway, triggering a series of metabolic adaptations in tumor cells, including changes in FAM [16,17]. Thus, elucidating the mechanisms and functional consequences of FAM reprogramming in CRC under hypoxic conditions is essential for understanding tumor adaptation within the complex microenvironment. This will not only improve our understanding of the metabolic regulatory network of CRC but also provide a theoretical basis for the development of novel targeted therapies.

Therefore, a comprehensive understanding is needed of how hypoxia reshapes FAM within both tumor cells and the surrounding microenvironment, and how these adaptations influence treatment response and differ between primary CRC and liver metastases. In this review, we first summarize the major steps of FAM reprogramming in CRC, including fatty acid uptake, *de novo* synthesis, FAO, lipid droplet dynamics, and key regulatory factors. We then focus on how hypoxic stress reshapes these processes through HIF-dependent and related adaptive pathways and examine how adipocytes, cancer-associated fibroblasts (CAFs), and immune-cell populations participate in FA metabolic crosstalk within the tumor microenvironment (TME). Finally, we discuss therapeutic strategies targeting FAM and hypoxia-associated signaling, including combinations with chemotherapy, ferroptosis induction, immunotherapy, and anti-angiogenic therapy. We also consider experimental models for studying hypoxia-associated FAM and fatty acid metabolic heterogeneity between primary CRC and liver metastases. Together, these perspectives provide a hypoxia-centered framework linking FAM to microenvironmental interactions, treatment response, and lesion-specific metabolic variation in CRC.

## 2. Literature Search Strategy and Study Selection

For this review, we performed a literature search in PubMed, Embase, Web of Science, and the Cochrane Library from database inception to 30 April 2026. The main search framework combined (“colorectal cancer” OR “colon cancer” OR “rectal cancer” OR “colorectal neoplasm”) with (“fatty acid metabolism” OR “lipid metabolism” OR “fatty acid uptake” OR “*de novo* lipogenesis” OR “fatty acid synthesis” OR “fatty acid oxidation” OR “beta-oxidation” OR “lipid droplet” OR “lipolysis” OR “CD36” OR “fatty acid transport protein” OR “FATP” OR “fatty acid-binding protein” OR “FABP” OR “ACLY” OR “ACC” OR “FASN” OR “SCD” OR “CPT1” OR “CPT2” OR “ACOX1” OR “HILPDA” OR “PLIN” OR “ATGL” OR “SREBP” OR “PPAR”). Searches focusing on the hypoxic tumor microenvironment additionally incorporated (“hypoxia” OR “hypoxia-inducible factor” OR “HIF-1α” OR “HIF1A” OR “HIF-2α” OR “EPAS1” OR “tumor microenvironment”). Additional targeted searches included terms related to microenvironmental interactions (“macrophage” OR “neutrophil” OR “myeloid-derived suppressor cell” OR “MDSC” OR “dendritic cell” OR “natural killer cell” OR “NK cell” OR “T cell” OR “adipocyte” OR “cancer-associated fibroblast” OR “CAF” OR “stromal cell” OR “immune cell” OR “lipid transfer” OR “metabolic crosstalk”), therapeutic strategies (“chemotherapy” OR “immunotherapy” OR “immune checkpoint blockade” OR “anti-angiogenic therapy” OR “bevacizumab” OR “ferroptosis”), experimental models (“two-dimensional culture” OR “tumor spheroid” OR “patient-derived organoid” OR “PDO” OR “co-culture” OR “organ-on-chip”), and metabolic heterogeneity (“primary tumor” OR “metastasis” OR “liver metastasis” OR “colorectal liver metastasis”).

We included studies that met one or more of the following criteria: (1) original studies investigating FAM, hypoxia, hypoxia-inducible signaling, or their interaction in CRC; (2) studies addressing fatty acid uptake, synthesis, desaturation, oxidation, lipid droplet dynamics, or lipolysis; (3) studies examining lipid metabolic interactions among tumor, stromal, and immune cells within the tumor microenvironment, including those occurring under hypoxic conditions; (4) preclinical, translational, or clinical studies evaluating FAM-related therapeutic targets or combination strategies; (5) studies using hypoxic cultures, tumor spheroids, patient-derived organoids, or co-culture models relevant to CRC; and (6) studies comparing lipid metabolic characteristics between primary and metastatic CRC.

We excluded (1) articles unrelated to CRC, FAM, hypoxia, or the tumor microenvironment; (2) studies lacking relevant mechanistic, therapeutic, clinical, or model-related information; (3) duplicate publications; (4) conference abstracts, editorials, or commentaries without sufficient original data; and (5) non-English articles. Two authors screened the titles and abstracts and subsequently assessed potentially eligible full-text articles. Disagreements were resolved through discussion, with consultation of a third author when necessary.

Original studies were prioritized and summarized in the main text and corresponding tables where appropriate. Guidelines, systematic reviews, meta-analyses, and selected narrative reviews were used to establish the broader clinical and methodological context. Because this article is a narrative review, no formal meta-analysis or risk-of-bias assessment was performed.

## 3. Mechanisms of Fatty Acid Metabolism Reprogramming in Colorectal Cancer

**Figure 1:**
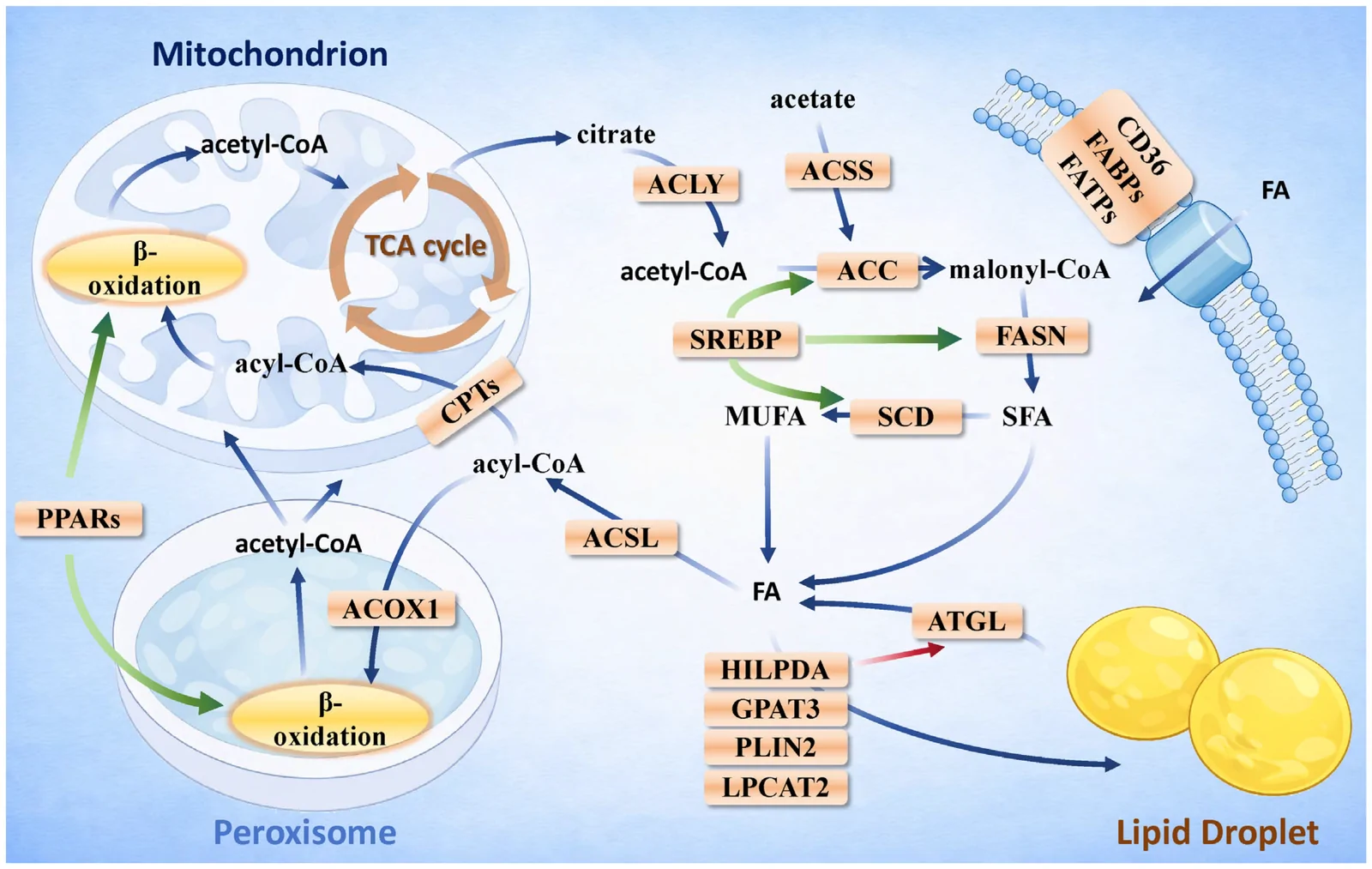
**Molecular mechanisms of fatty acid metabolism reprogramming in colorectal cancer.** Blue arrows indicate metabolic conversion or transport, green arrows indicate positive regulation, and red arrows indicate inhibitory regulation. Abbreviations: ACC, acetyl-CoA carboxylase; acetyl-CoA, acetyl coenzyme A; ACLY, ATP-citrate lyase; ACOX1, acyl-CoA oxidase 1; ACSL, acyl-CoA synthetase long-chain family; ACSS, acetyl-CoA synthetase; acyl-CoA, acyl coenzyme A; ATGL, adipose triglyceride lipase; CD36, cluster of differentiation 36; CPT, carnitine palmitoyltransferase; FA, fatty acid; FABP, fatty acid-binding protein; FASN, fatty acid synthase; FATP, fatty acid transport protein; GPAT3, glycerol-3-phosphate acyltransferase 3; HILPDA, hypoxia-inducible lipid droplet-associated protein; LPCAT2, lysophosphatidylcholine acyltransferase 2; malonyl-CoA, malonyl coenzyme A; MUFA, monounsaturated fatty acid; PLIN2, perilipin 2; PPAR, peroxisome proliferator-activated receptor; SCD, stearoyl-CoA desaturase; SFA, saturated fatty acid; SREBP, sterol regulatory element-binding protein; TCA, tricarboxylic acid cycle.

FAs are carboxylic acids involved in energy storage, derived from dietary intake or endogenous synthesis from acetyl coenzyme A (acetyl-CoA). Exogenous FAs can be stored as triglycerides and cholesterol esters. Saturated fatty acids (SFAs) contain no unsaturated double bonds, whereas FAs with unsaturated bonds in their hydrocarbon chain are termed unsaturated fatty acids (UFAs). These include monounsaturated fatty acids (MUFAs), containing one unsaturated bond, and polyunsaturated fatty acids (PUFAs), containing more than one unsaturated bond [18,19]. FAs are present in various cellular structures and participate in signal transduction and energy storage. Normal cells typically rely on exogenous FAs, whereas malignant cells can activate FAM pathways to increase FA synthesis. The major pathways of FAM reprogramming in CRC are illustrated in Figure 1, and the corresponding enzymes and regulatory factors are summarized in Table 1.

### 3.1. Fatty Acid Uptake

Cellular FA availability is maintained through endogenous synthesis, passive diffusion, and transporter-mediated uptake of exogenous FAs, which together support cellular energy demand. Tumor cells can regulate FA uptake based on metabolic requirements in order to meet their high energy demands. Exogenous FAs taken up by tumor cells can be stored in lipid droplets (LDs), which serve to maintain lipid homeostasis, prevent lipotoxicity, and provide a source of ATP and reduced nicotinamide adenine dinucleotide phosphate (NADPH) under conditions of metabolic stress [72]. Furthermore, the uptake of exogenous FAs sustains lipid requirements under metabolic stress conditions such as hypoxia [73]. Proteins involved in FA transport include fatty acid transport proteins (FATPs), fatty acid-binding proteins (FABPs), and fatty acid translocase (FAT/CD36), which regulate FA uptake, trafficking, storage, and signaling [15,74,75].

FABPs constitute a multifunctional protein family involved in FAM. Twelve subtypes operate as intracellular FA carriers, and their expression has been connected to the development of certain tumors [23]. FABP1 and FABP6 are significantly upregulated in CRC, whereas elevated FABP4 and FABP6 expression is associated with poor prognosis [21]. Moreover, FABP4 expression is higher in stage III–IV than in stage I–II CRC and is associated with lymph node metastasis [24]. Mechanistically, FABP4 promotes intracellular lipid accumulation and activates AKT-dependent epithelial–mesenchymal transition (EMT), invasion, and metastasis in colon cancer [22]. In metastatic CRC cells, FABP5 upregulation is sustained by promoting DNA demethylation and NF-κB pathway activation. Additionally, knockdown of *FABP5* can lead to cell cycle arrest in the G1 phase and induce apoptosis [20].

FAT, also known as CD36, is a membrane glycoprotein receptor found on the surfaces of many cells that can stimulate the growth and progression of multiple tumors [27,76]. CD36 does not appear to function uniformly in CRC, and its effects seem to vary across biological settings. Although reduced CD36 expression has been reported in some settings, accumulating evidence links CD36 to malignant transformation and poor prognosis in CRC [25,30]. On one hand, CD36 may exert tumor-suppressive effects through inhibition of downstream aerobic glycolysis [28]. At the same time, CD36-mediated lipid uptake may promote CRC cell proliferation and metastasis by increasing the availability of exogenous FAs [26,29]. Furthermore, CD36 contributes to CRC metastasis, at least in part, by increasing matrix metalloproteinase 28 (MMP28) [27]. Taken together, although CD36 may exert distinct effects in specific metabolic contexts, current evidence more strongly supports its pro-tumor role in lipid uptake, metastatic progression, and poor prognosis in CRC.

The FATP family consists of integral membrane proteins that facilitate FA uptake through extracellular FA-binding activity and play diverse roles in FAM. FATPs are frequently overexpressed in cancers, where they enhance FA uptake and contribute to tumor growth or invasion [74,77,78]. *FATP5* expression is higher in CRC tissues than in non-tumor tissues, whereas relatively low intratumoral *FATP5* expression is associated with poorer survival among patients with CRC. Consistently, *FATP5* knockdown increased the proliferation of CACO-2 and HCT116 cells and reduced their accumulation in the G2/M phase, supporting a growth-restraining role of *FATP5* in CRC cells [31]. Collectively, enhanced FA uptake in CRC is mediated by multiple transport-related proteins, which support tumor growth, metastatic dissemination, and treatment adaptation by increasing lipid availability.

### 3.2. De Novo Fatty Acid Synthesis

In addition to taking up exogenous FAs, CRC cells can activate endogenous lipogenic pathways to increase FA availability under metabolic stress in the TME. Acetyl-CoA, produced in mitochondria from citrate via the tricarboxylic acid (TCA) cycle, serves as the substrate for *de novo* FA synthesis [79,80]. Citrate is then transported to the cytoplasm, where it is converted back to acetyl-CoA for *de novo* synthesis by ATP-citrate lyase (ACLY) [81]. Under metabolic stress, such as hypoxia or lipid-depleted conditions, cancer cells can utilize acetate to produce acetyl-CoA via upregulation of acetyl-CoA synthetase (ACSS), circumventing the need for citrate [82,83]. Acetyl-CoA is first carboxylated to malonyl-CoA by acetyl-CoA carboxylase (ACC), after which fatty acid synthase (FASN) catalyzes the sequential condensation reactions required for SFA synthesis [19,84]. Stearoyl-CoA desaturase (SCD) can then desaturate SFAs to produce MUFAs [42,43].

ACLY is the first rate-limiting enzyme of *de novo* synthesis, catalyzing the conversion of mitochondrial-derived citrate into acetyl-CoA. ACLY plays a central role in cancer-associated FAM through multiple mechanisms. ACLY promotes CRC progression by stabilizing β-catenin, and its expression and metastatic potential are associated with chemotherapy resistance [33]. Another study showed that homeobox A13 (HOXA13) promotes CRC metastasis by activating ACLY and insulin-like growth factor 1 receptor (IGF-1R) signaling [32].

ACC exists in two isoforms and serves as the rate-limiting enzyme for FA synthesis, regulating lipogenesis, metastasis, and apoptosis in tumor cells. Research indicates that AMPK regulates ACC activity primarily through direct phosphorylation [34,84]. Regenerating gene 4 (REG4) promotes proteasomal degradation of ACC1 and ACLY while enhancing LD assembly and chemoresistance in CRC [36]. LncRNA *CRCMSL* can suppress CRC metastasis by limiting ACC activity and reducing membrane fluidity [35].

FASN is an enzyme that converts acetyl-CoA and malonyl-CoA to FAs, directing carbohydrates toward FA synthesis, which can subsequently be stored or oxidized for energy. FASN activity is relatively low in normal tissues but is elevated in tumors because of the increased cellular demand for endogenous SFA synthesis. FASN expression is markedly elevated in CRC and enhances lipogenesis and ATP production, thereby supporting tumor growth and metastatic potential [38,39,40]. FASN promotes β-catenin signaling, enhancing the stem-like characteristics of CRC cells [37].

SCD is an important desaturase in *de novo* synthesis that catalyzes the conversion of SFAs such as palmitate and stearate into MUFAs, which supports membrane biosynthesis and tumor metabolism. Simultaneous activation of glycolysis and FA synthesis in cancer cells promotes SCD-dependent MUFA production, which supports rapid tumor growth and metastasis. High SCD1 expression is associated with malignant progression and promotes EMT in CRC [41,42]. Nodal overexpression can activate *SCD1* transcription via Smad2/3 signaling, resulting in MUFA production in CRC [43]. Overall, *de novo* synthesis provides CRC cells with both structural lipids and metabolic flexibility, particularly when nutrients or oxygen are limited.

### 3.3. Fatty Acid Oxidation

Tumor cells can adapt to nutrient-deficient microenvironments by enhancing FAO to meet the increased energy demands for growth. FAO involves FA activation, transport, β-oxidation, and coupling to the TCA cycle. In CRC, β-oxidation contributes to tumor development and occurs in both mitochondria and peroxisomes [44,49]. Cytosolic FAs are first activated by acyl-CoA synthetase long-chain (ACSL) family members to form acyl-CoAs. Acyl-CoAs are then converted to acylcarnitines by carnitine palmitoyltransferase 1 (CPT1) for transport into mitochondria. Carnitine palmitoyltransferase 2 (CPT2) in mitochondria regenerates acyl-CoA, which is then converted to acetyl-CoA through β-oxidation. Acetyl-CoA subsequently enters the TCA cycle, along with oxidative phosphorylation, to generate ATP and NADPH, which provide energy and reducing power for tumor growth and redox homeostasis. ATP fuels tumor growth, whereas NADPH is required for anabolic activities and reactive oxygen species (ROS) detoxification. In hypoxic tumor cells, FA uptake and LD accumulation increase, providing sufficient ATP for recovery during reoxygenation, while elevated NADPH levels help protect cells from ROS-induced damage [16]. Acyl-CoA oxidase 1 (ACOX1) catalyzes the initial oxidation step of peroxisomal FAO and contributes to acyl-CoA oxidation [49]. FAO exerts diverse effects in tumor cells; it can provide energy for growth and migration, yet under specific conditions it may also restrain tumor progression through enhanced FA consumption. This complex mechanism depends on the cell type, metabolic environment, and cancer stage.

In CRC, the role of FAO appears to be context-dependent rather than uniformly pro-tumor or tumor-suppressive. In many settings, FAO supports tumor growth, survival, and metastatic adaptation by preserving energy supply and redox balance. However, under specific conditions, FAO-associated pathways may also restrain malignant progression when linked to lower stemness, weaker glycolytic rewiring, or greater treatment sensitivity [46,49,85]. Overall, the functional impact of FAO in CRC is shaped by metabolic context, tumor state, and treatment pressure. The CPT system consists of CPT1 and CPT2. CPT1 is situated on the outer mitochondrial membrane, while CPT2 is distributed on the inner membrane. CPT1 serves as the rate-limiting enzyme in FAO and regulates this pathway in many tumors [44,45]. Malonyl-CoA, generated during FA synthesis, inhibits CPT1 activity; accordingly, ACC inhibition reduces malonyl-CoA levels and promotes FAO [84]. Activation of CPT1A-mediated FAO suppresses anoikis in CRC cells, improving metastatic potential [44]. CPT1C expression raises the FAO rate and promotes CRC proliferation; however, downregulating CPT1C expression via RNA interference greatly reduces the FAO rate, inhibits tumor cell proliferation, and induces cell cycle arrest in G1 [45]. CPT2 expression is frequently downregulated in CRC, and CPT2 loss promotes proliferation and inhibits p53-dependent apoptosis [47]. Conversely, downregulation of CPT2 can enhance CRC cell stemness and chemoresistance by activating β-catenin-induced glycolytic metabolism [46].

The acyl-CoA oxidase (ACOX) family comprises three isoforms (ACOX1–3) and plays a critical role in peroxisomal FAO, particularly in the oxidation of very-long-chain fatty acids (VLCFAs) [86]. In CRC, ACOX1 downregulation promotes tumor cell proliferation and migration, whereas ACOX1 overexpression suppresses tumor growth [48,49]. Therefore, the role of peroxisomal FAO in tumors warrants further exploration. ACOX1 expression and FAO activity are inversely associated with solute carrier family 9 member A5 (*SLC9A5*). *SLC9A5* promotes tumor growth and metastasis by negatively regulating ACOX1, which inhibits its FA oxidation activity [49].

### 3.4. Lipid Droplet Biology and Lipolysis

CRC cells rely on LDs to regulate FA supply and demand in response to metabolic alterations and stress conditions. LDs are organelles that store neutral lipids, mainly triglycerides and cholesteryl esters, and mobilize stored FAs in response to increased cellular energy demand [87,88]. LDs support cellular energy balance and metabolic adaptation by regulating FA storage, release, and oxidation. The extent of FA accumulation in CRC correlates with tumor aggressiveness. Various enzymes regulate FA storage in LDs, and specific lipases can hydrolyze triglycerides within LDs to release FAs [89,90]. The synthesis and breakdown of LDs are precisely regulated by multiple mechanisms to meet the lipid demands of tumor cells under different conditions [87]. Furthermore, LD formation serves as a protective lipid-storage mechanism under nutrient-deprived conditions such as tumor hypoxia. In hypoxic environments, LDs store FAs and facilitate mitochondrial remodeling, thereby enabling cells to generate energy through lipid β-oxidation [16].

LDs contribute to CRC progression by supporting metabolic adaptation, particularly in the context of chemoresistance. Beyond serving as lipid storage organelles, LDs also function as metabolic buffers that connect stress adaptation with treatment response in CRC. In chemotherapy-exposed tumor cells, increased LD formation can reduce lipotoxicity and endoplasmic reticulum stress, thereby helping cells tolerate cytotoxic injury and favoring drug resistance [51,90,91]. LD-associated remodeling may also influence the immune microenvironment, as altered lipid handling has been linked to impaired antitumor immunity and enhanced immune escape [92,93]. In addition, LD metabolism appears to be closely connected to ferroptosis [94]. Because ferroptosis depends on iron-driven lipid peroxidation, the balance between PUFA storage, phospholipid remodeling, and LD turnover may affect whether CRC cells are protected from or sensitized to ferroptotic death [95]. These observations suggest that LDs are not merely passive lipid reservoirs, but active participants linking metabolic adaptation, therapeutic resistance, immune regulation, and ferroptosis-related vulnerability in CRC. This broader role of LDs is increasingly reflected in studies of CRC-associated enzymes involved in LD biogenesis, phospholipid remodeling, lipolysis, and hypoxia-responsive lipid storage. Glycerol-3-phosphate acyltransferase 3 (GPAT3) is an essential lipid metabolic enzyme that catalyzes the formation of lysophosphatidic acid (LPA), which then initiates triacylglycerol synthesis and LD formation. Overexpression of GPAT3 in CRC promotes tumor cell proliferation while increasing chemoresistance through enhanced LD formation [50]. Lysophosphatidylcholine acyltransferase 2 (LPCAT2) is an LD-localized enzyme that promotes phosphatidylcholine production. It regulates LD accumulation in CRC cells by buffering FAs generated during lipolysis, protecting mitochondria from lipotoxicity, and thereby supporting chemoresistance [51]. SCD1 can also increase FA synthesis and LD accumulation, which helps CRC cells survive. Inhibiting SCD1 can activate the AMPK pathway, which promotes lipolysis and FA degradation, decreasing LD accumulation and increasing CRC cell chemosensitivity [54]. Moreover, SCD1 inhibition may enhance antitumor immunity by promoting CD8^+^ T cell activation and interferon gamma (IFN-γ) production and may synergize with anti-programmed cell death protein 1 (PD-1) therapy [52,53]. Furthermore, overexpression of FABPs promotes LD accumulation and chemoresistance in CRC cells. Krüppel-like factor 5 (KLF5) promotes FABP6 transcription, increasing LD formation and CRC cell proliferation, and is closely associated with resistance to chemotherapeutic agents such as oxaliplatin [55]. Conversely, tripartite motif-containing 3 (TRIM3) can promote the ubiquitin-mediated degradation of FABP4, reducing LD formation and inhibiting the migration and invasion of CRC cells [56].

Adipose triglyceride lipase (ATGL) is a rate-limiting enzyme in triglyceride degradation and strongly influences the ability of cells to utilize FAs as an energy source. ATGL is elevated in CRC and may promote tumor progression by enhancing triglyceride degradation. Obesity may promote ATGL-mediated LD utilization in CRC [57,58]. Hypoxia-inducible lipid droplet-associated (HILPDA) protein is induced by hypoxia through hypoxia-inducible factor 1α (HIF-1α) binding to hypoxia-response elements upstream of its transcription start site. HILPDA expression is elevated in CRC, and FAs can both induce its expression and inhibit its degradation [60]. HILPDA is an important regulator of cancer progression by inhibiting ATGL-mediated intracellular lipolysis [59]. HILPDA deficiency reduces LD and triglyceride stores in CRC, an effect that can be reversed by ATGL inhibition. Perilipins (PLINs), also known as adipose differentiation-related proteins (ADRPs), are essential for LD formation and the maintenance of lipid homeostasis. This family comprises five isoforms (PLIN1–5). PLIN2 is upregulated in CRC and may serve as a potential biomarker for early-stage disease [61]. PLIN2 can promote CD36-mediated EMT and accelerate CRC progression [62]. Collectively, these findings place LDs at the intersection of metabolic adaptation, therapy resistance, immune modulation, and ferroptosis-related vulnerability in CRC.

### 3.5. Key Regulators of Fatty Acid Metabolism

Recent research has found that a variety of factors influence the activation or inhibition of FAM enzymes, regulating tumor growth and metastasis. Sterol regulatory element-binding proteins (SREBPs) are important transcription factors for lipid metabolism that are upregulated in CRC. They regulate the synthesis of FAs, triacylglycerols, and cholesterol and thus contribute to tumorigenesis. SREBP1 and SREBP2 principally control the synthesis of FAs and cholesterol, respectively. SREBPs drive CRC proliferation and metastasis by modulating the expression of lipogenic genes such as FASN, SCD, and ACC1 [63,66]. Prothymosin α (PTMA) promotes SREBP1-mediated expression of key enzymes such as FASN and SCD1, enhancing FA synthesis and LD accumulation. This leads to increased chemotherapy resistance in CRC cells [64]. Zinc finger antisense 1 (*ZFAS1*) can also enhance the expression of FASN and SCD1 by regulating SREBP1 expression, consequently promoting lipid accumulation and tumor proliferation in CRC [65].

Peroxisome proliferator-activated receptors (PPARs) are key regulators of lipid metabolic balance and energy homeostasis. The PPAR family comprises three subtypes: α, β/δ, and γ. CRC tissues exhibit increased expression of PPARα and PPARβ/δ, accompanied by reduced expression of PPARγ. PPARs may function as either tumor suppressors or oncogenic regulators depending on the biological context [69]. PPARα appears to have context-dependent effects in CRC. Although it may suppress CRC through regulation of glucose metabolism and mTOR signaling, PPARα has also been reported to promote proliferation and invasion by enhancing FAO and LD accumulation, particularly in acidic microenvironments [67,68]. Protein tyrosine phosphatase receptor type O (PTPRO) activates PPARα, leading to increased production of the FAO enzyme ACOX1, which facilitates tumor proliferation. Activation of the AKT/mTOR signaling pathway leads to downregulation of PPARα, which reduces its influence on FAO. Interestingly, PTPRO can also limit FA synthesis by inhibiting SREBP1 expression and subsequently downregulating ACC1 activity. PPARβ/δ can enhance FAO. A high-fat diet activates PPARβ/δ and PPARα, increasing CPT1A-mediated FAO in intestinal stem cells and enhancing their tumorigenicity [70]. PPARγ, a lipid metabolism-related nuclear receptor, is widely expressed in various adipose tissues [96,97]. ZDHHC6-mediated palmitoylation stabilizes PPARγ, activates downstream enzymes such as ACLY, and promotes FA synthesis [71].

Furthermore, the regulatory roles of other factors on FAM-related enzymes are gradually being uncovered. REG4 reduces *de novo* synthesis and affects LD assembly by transcriptionally repressing ACC1 and ACLY and promoting their proteasomal degradation. Despite this inhibitory effect on lipogenesis, REG4 has also been linked to increased LD accumulation and chemoresistance in CRC cells, suggesting that its impact on lipid remodeling may extend beyond *de novo* synthesis alone [36]. KLF5 promotes CRC progression and drug resistance by upregulating FABP6 transcription [55].

## 4. Hypoxia and Fatty Acid Metabolic Reprogramming in the CRC Microenvironment

**Figure 2:**
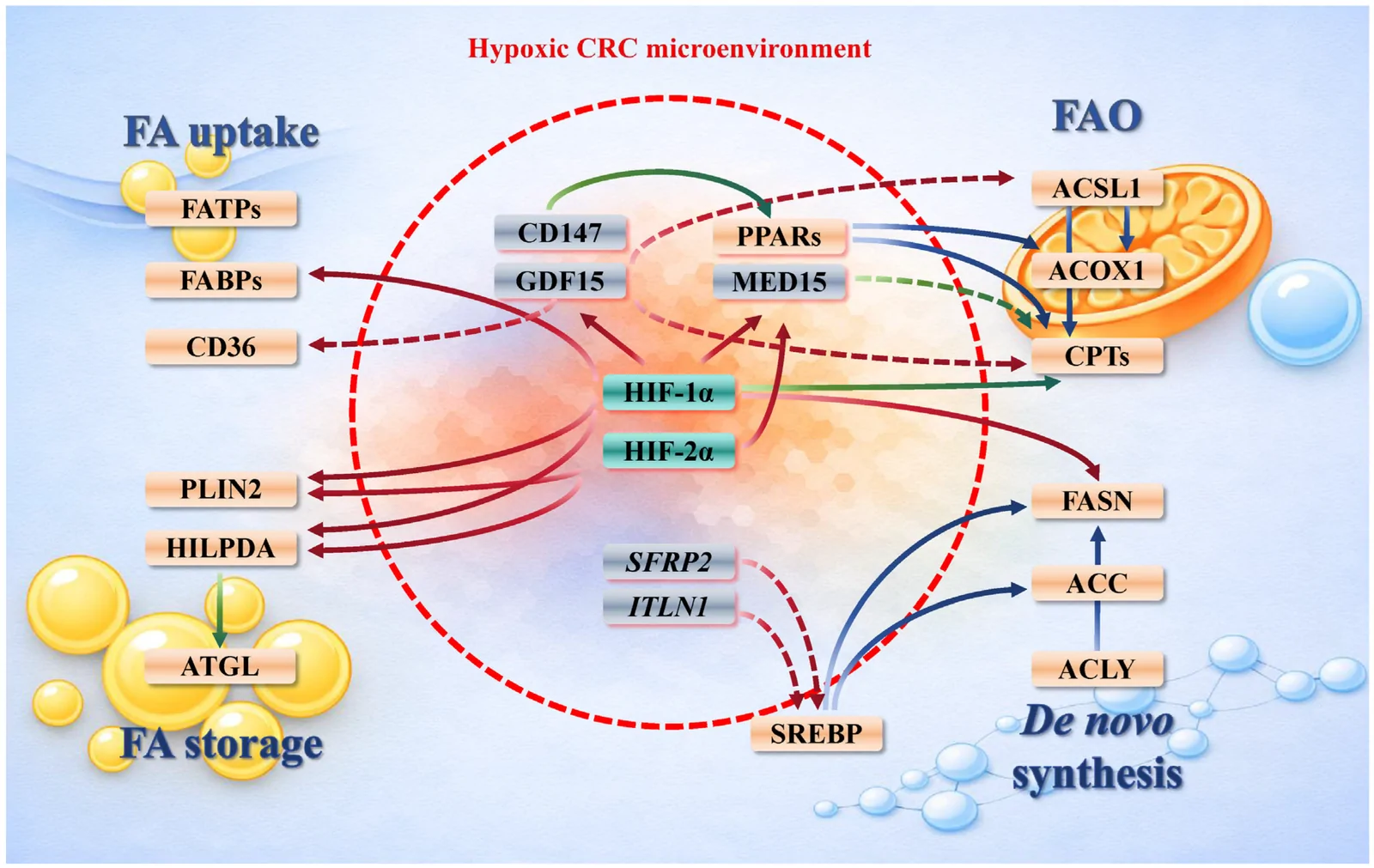
**Hypoxia-driven regulation of fatty acid metabolism in colorectal cancer.** Red and green arrows denote hypoxia-enhanced and hypoxia-suppressed regulation, respectively, whereas blue arrows indicate the direction of normal fatty acid metabolism. Solid and dashed arrows indicate HIF-dependent and HIF-independent hypoxic regulation, respectively. The red dashed circle delineates the hypoxic CRC microenvironment. Abbreviations: ACC, acetyl-CoA carboxylase; ACLY, ATP-citrate lyase; ACOX1, acyl-CoA oxidase 1; ACSL1, acyl-CoA synthetase long-chain family member 1; ATGL, adipose triglyceride lipase; CD147, cluster of differentiation 147; CD36, cluster of differentiation 36; CPT, carnitine palmitoyltransferase; CRC, colorectal cancer; FA, fatty acid; FABP, fatty acid-binding protein; FAO, fatty acid oxidation; FASN, fatty acid synthase; FATP, fatty acid transport protein; GDF15, growth differentiation factor 15; HIF-1α, hypoxia-inducible factor 1α; HIF-2α, hypoxia-inducible factor 2α; HILPDA, hypoxia-inducible lipid droplet-associated protein; *ITLN1*, intelectin 1; MED15, mediator complex subunit 15; PLIN2, perilipin 2; PPAR, peroxisome proliferator-activated receptor; *SFRP2*, secreted frizzled-related protein 2; SREBP, sterol regulatory element-binding protein.

Rapid tumor cell proliferation often outpaces local angiogenesis, leading to restricted oxygen and nutrient supply and the development of a persistent hypoxic microenvironment within and around the tumor [98]. Hypoxia profoundly influences CRC FA uptake, synthesis, oxidation, and storage, thereby reshaping the metabolic network of CRC. Hypoxia-mediated FAM reprogramming is central to maintaining CRC cell energy homeostasis, promoting survival, invasion, metastasis, and therapeutic resistance [45,99,100,101]. In parallel, adipocytes, CAFs, and immune cells regulate lipid availability, transfer, and utilization within the CRC microenvironment [29,92,102]. The influence of hypoxia on these interactions is clearer for macrophages and remains less defined for adipocytes and CAFs [103]. The major hypoxia-driven changes in FAM in CRC cells are illustrated in Figure 2. The corresponding regulators in CRC cells and macrophages are summarized in Table 2.

### 4.1. Hypoxia and Fatty Acid Metabolic Features in CRC

Hypoxia is a defining feature of the tumor microenvironment in CRC and a major driver of metabolic adaptation. Under low-oxygen conditions, CRC cells remodel FAM to support survival, proliferation, and stress tolerance. This metabolic shift contributes to tumor aggressiveness and therapeutic resistance [99,101].

Although glycolytic reprogramming is a classic response to hypoxia, hypoxic tumor cells can also reshape FAM to preserve energy homeostasis. The HIF pathway is central to this process, with HIF-1α promoting FA uptake and lipid storage while modulating FAO and lipolysis [16,60,104]. In addition to HIF-dependent responses, tumor cells activate multiple non-HIF pathways to adapt to hypoxic stress. These include mechanisms that sense nutrient and energy availability, such as mTORC1 and AMPK, stress-response programs including the unfolded protein response (UPR) and integrated stress response (ISR), and oxidative stress-mediated signaling [108,109,110,111]. Collectively, these pathways help tumor cells maintain protein and organelle homeostasis, coordinate metabolic and biosynthetic demands, manage redox balance, and survive under low-oxygen conditions.

In CRC, hypoxia directly reshapes FA uptake, synthesis, oxidation, and storage, thereby creating a flexible metabolic program rather than a single uniform response [100,104,112]. These changes support energy balance, limit lipotoxic and oxidative stress, and promote invasion, metastasis, and therapy resistance. Together, HIF-dependent and non-HIF mechanisms form a multifaceted regulatory network linking hypoxia to FAM reprogramming in CRC.

### 4.2. Interaction Between HIF Signaling and Fatty Acid Metabolism in CRC

Among the pathways that mediate hypoxic adaptation, HIF signaling represents the central mechanism linking low-oxygen stress to FAM reprogramming. In CRC, the HIF pathway supports tumor cell survival and metabolic adaptation under hypoxic conditions by promoting FA synthesis and selectively remodeling FAO. Hypoxia-induced HIF-1α enhances lipid uptake, endogenous synthesis, and lipid storage through upregulation of FABPs, PLIN2, FASN, and SREBP1, while suppressing certain FAO components such as CPT1 [104]. At the same time, HIF-1α can transcriptionally activate CPT1C, which increases FAO activity and promotes proliferation, S-phase progression, and migration. High CPT1C expression is associated with poorer relapse-free survival in patients with CRC [45]. Notably, FAO is closely involved in hypoxia adaptation. Acetyl-CoA generated by FAO not only enters the TCA cycle for energy production but can also act as a precursor, indirectly promoting glycogen synthesis. Under hypoxic conditions, CRC cells rely on increased FAO for glycogen synthesis, which provides energy for invasion and metastasis. In vitro spheroid models, which more closely mimic the oxygen environment of solid tumors, showed that heme oxygenase 1 (*HMOX1*) induction depends on FAO activity, whereas glucose transporter 1 (*GLUT1*) expression does not [112]. These findings suggest that FAO not only supports cell survival under hypoxic conditions but also promotes malignant phenotypes through metabolic regulation. Under hypoxic conditions, elevated HILPDA causes a rise in LDs, which buffers lipotoxicity and contributes to the stability of cellular energy metabolism [60]. 

HIF-1α effector molecules also participate in the regulation of metabolic adaptation. Growth differentiation factor 15 (GDF15), a downstream effector of HIF-1α, promotes FAO by upregulating CPT1, CPT2, ACSL1, and CD36 [101]. This increases energy availability and facilitates CRC cell proliferation and migration. Conversely, FA metabolism-related proteins can also function upstream of HIF-1α. FABP5 promotes CRC cell proliferation by activating the HIF-1α signaling pathway. Using a 3D culture-chip model, researchers showed that *FABP5* knockdown under hypoxic conditions markedly reduced HIF-1α protein levels and downstream target gene expression and partially reversed hypoxia-associated effects [113]. Cluster of differentiation 147 (CD147) further links HIF-1α signaling with glycolipid metabolic reprogramming by activating PI3K/AKT/mTOR-dependent HIF-1α-mediated glycolysis while suppressing PPARα-mediated FAO through the MAPK pathway, thereby contributing to 5-fluorouracil (5-FU) resistance in CRC [99]. Hypoxia-inducible factor 2α (HIF-2α) also regulates oxidative stress and FAM in CRC. In the hypoxic TME, HIF-2α regulates HILPDA and PLIN2, thereby modulating oxidized lipid accumulation, ferroptosis-related responses, and tumor cell behavior [105]. In addition, hypoxia promotes LD accumulation through HIF-1α/HIF-2α–mediator complex subunit 15 (MED15) signaling, which suppresses CPT1A expression [100].

### 4.3. HIF-Independent Regulation of Fatty Acid Metabolism in CRC Under Hypoxia

Beyond HIF-dependent signaling, hypoxic adaptation in CRC may also reshape FAM through several non-HIF pathways. These include regulators involved in cellular responses to nutrient and oxygen availability, such as PPARα, stress-response pathways like the UPR and mTORC1-dependent signaling, and oxidative stress-mediated modulation of lipid synthesis and FAO [67,114,115,116]. Alongside these broader programs, some hypoxia-responsive genes contribute additional fine-tuning to lipid metabolic adaptation. Together, these mechanisms create a multifaceted regulatory system by which hypoxia directs fatty acid synthesis, oxidation, storage, and overall metabolic plasticity in CRC. One study used bioinformatics analysis to identify hypoxia- and lipid metabolism-related genes and found upregulation of secreted frizzled-related protein 2 (*SFRP2*) and downregulation of intelectin 1 (*ITLN1*) in CRC cell lines under hypoxia. *SFRP2* and *ITLN1* are involved in CRC cell proliferation, migration, EMT, and responses to immunotherapy. Hypoxia modulates SREBP1 expression through *SFRP2* and *ITLN1*, which may enhance lipid metabolic reprogramming and promote CRC progression [106]. Furthermore, in the acidic environment following hypoxia, PPARα stimulates the generation of important enzymes such as ACOX1, which enhance mitochondrial and peroxisomal FAO capability. This allows cancer cells to use FAs as an energy source while surviving in the acidic microenvironment [107]. Certain hypoxia-responsive genes, such as *HMOX1*, require FAO for activation, while *GLUT1*, a classic HIF-1α target, does not. This implies that the HIF signaling system and FAM are not linked in a simple linear manner. Instead, FAO supports certain hypoxia-responsive pathways, hence changing the metabolic variability and invasive potential of cancers in the hypoxic environment [112]. Despite these advances, additional non-HIF hypoxia-adaptive pathways and their interactions with FAM remain poorly understood, highlighting the need for further investigation.

### 4.4. Fatty Acid Metabolic Crosstalk with Stromal and Immune Cells

Adipocytes, CAFs, and immune cells influence CRC progression through lipid supply, metabolic adaptation, and intercellular lipid transfer. The principal changes in fatty acid metabolism involving these cells are summarized in Table 3. Beyond lipid metabolism intrinsic to tumor cells, adipocytes and metabolically specialized CAF subsets regulate extracellular lipid availability and reshape fatty acid uptake, storage, and utilization in CRC cells [22,117]. Adipocytes isolated from patients with colon cancer have been shown to release free fatty acids that are transferred to adjacent cancer cells, thereby increasing mitochondrial fatty acid β-oxidation, inducing autophagy through AMPK activation, and supporting cancer cell survival under nutrient deprivation [102]. CAFs can similarly provide a metabolic niche rich in lipids. Compared with normal fibroblasts, CAFs isolated from CRC tissues exhibit increased fatty acid and phospholipid abundance together with elevated FASN expression; lipids from CAFs are subsequently taken up by CRC cells through CD36, promoting cancer cell migration and metastatic capacity [29]. Stable isotope tracing analyses further demonstrated that fatty acids supplied by CAFs are incorporated into unsaturated phosphatidylcholines in CRC cells, increasing plasma membrane fluidity and facilitating peritoneal dissemination [118]. In addition to supplying lipids, CAFs can adapt their fatty acid utilization: under glucose limitation, increased CPT1A activity supports FAO, thereby sustaining CAF survival and promoting CRC peritoneal metastasis [119]. Collectively, adipocytes and specific CAF subsets contribute to the regulation of fatty acid availability, uptake, incorporation, and oxidation within the CRC microenvironment.

CRC cells can also reprogram adipocytes and fibroblasts toward stromal states that support tumor progression. In co-cultures with primary human adipocytes, CRC cells induced a phenotype resembling cancer-associated adipocytes, characterized by dedifferentiation toward a fibroblast phenotype, LD redistribution, reduced expression of proteins involved in adipogenesis and lipid handling, and a shift from mitochondrial respiration toward glycolytic ATP production [120]. Signals from CRC cells also generate functionally distinct CAF states. Oncogenic KRAS signaling through transcription factor CP2 (TFCP2) reprogrammed fibroblasts into CAFs with high lipid content and increased vascular endothelial growth factor A (VEGFA) production, thereby promoting angiogenesis and CRC progression [122]. Exosomes from CRC cells reprogrammed CAFs derived from hepatic stellate cells through ACLY activation and increased C-X-C motif chemokine ligand 5 (CXCL5) secretion, supporting a prometastatic liver niche [123]. Palmitic acid accumulation activated NF-κB signaling and cytokine production in CRC cells, thereby promoting the formation of CAFs with increased matrix production and extracellular matrix stiffening [124]. Exposure to adipocytes increased FABP4 expression in CRC cells exhibiting drug tolerance, whereas FABP4 inhibition restored cetuximab sensitivity [121]. Together, interactions between CRC cells, adipocytes, and CAFs remodel stromal lipid metabolism and contribute to angiogenesis, metastatic niche formation, matrix remodeling, and treatment resistance.

FAM also shapes the functional states of immune cell populations in CRC. In tumor-associated macrophages, LD formation and subsequent FAO sustain mitochondrial respiration and an immunosuppressive phenotype, whereas inhibition of LD formation or FAO attenuates these effects [92]. In CRC, V-set and immunoglobulin domain-containing 4 (VSIG4) promotes M2-like macrophage polarization through FAO, thereby facilitating immune escape [125]. In the liver metastatic niche, interleukin-33 induces lipid droplet formation in mature low-density neutrophils, maintaining their survival and immunosuppressive activity. These neutrophils transfer lipids to dormant CRC cells, enhance FAO and eicosanoid synthesis, and facilitate metastatic reactivation [93]. FA composition can also alter the balance between suppressive and effector immune populations. In CRC models, a high-fat diet and palmitic acid increased myeloid-derived suppressor cell accumulation and reduced CD8^+^ T cell infiltration, whereas conjugated linoleic acid restored antitumor immune activity [127]. Conversely, SCD1 inhibition increased dendritic cell recruitment and antitumor CD8^+^ T cell responses, reduced endoplasmic reticulum stress in T cells, and enhanced the efficacy of anti-PD-1 treatment in MC38 and CT26 models [52].

Within the CRC microenvironment, hypoxia reprograms macrophage lipid metabolism and alters their interactions with tumor and immune cells. In hypoxic macrophages, colony-stimulating factor 1 receptor inhibition reduced lipid biosynthesis and destabilized HIF-2α, thereby decreasing dihydropyrimidine dehydrogenase (DPD) expression and restoring CRC sensitivity to 5-FU [126]. In the premetastatic liver, hypoxia induced HIF-1α, which upregulated FABP7 in macrophages, leading to lipid droplet formation through diacylglycerol acyltransferase 1 (DGAT1), increased FAO, and M2-like polarization. These macrophages accumulated abundant lipids and transferred exosomes rich in triglycerides to CRC cells and CD8^+^ T cells, increasing FAO in tumor cells while impairing T cell metabolism and effector function [103]. Lipid remodeling under hypoxia also affects immune surveillance by NK cells. In a co-culture model under hypoxia, CRC stem cells derived from HT-29 cells reduced NK cell cytotoxicity and altered cytokine secretion through docosadienoic acid metabolism involving fatty acid desaturase 1 (FADS1) [128]. Hypoxia also induces Wnt5a secretion by fibroblasts, which promotes colon cancer progression [17]. The effects of hypoxia on adipocyte lipolysis, CAF lipid synthesis and oxidation, and fatty acid exchange with other immune cell populations remain to be characterized.

## 5. Therapeutic Targeting of Fatty Acid Metabolism and Hypoxia-Related Pathways in CRC

Based on the mechanisms discussed above, targeting FAM and hypoxia-related pathways has emerged as a promising therapeutic strategy for CRC. The synergistic interplay between FAM reprogramming and the hypoxic TME not only enhances metabolic flexibility and survival in tumor cells but also markedly increases their resistance to chemotherapy. Thus, inhibiting key FAM enzymes may disrupt tumor metabolic homeostasis and help overcome drug resistance. Concurrently, therapies targeting hypoxia and its associated signaling pathways offer a promising avenue for overcoming microenvironment-driven drug resistance. Representative therapeutic and combination strategies targeting FAM are summarized in Table 4.

### 5.1. Targeting Fatty Acid Uptake

Tumor cell metabolism is characterized by a high demand for FAs, which are employed not only for membrane biosynthesis but also as an energy source. Targeting FA uptake routes provides novel treatment options for CRC. CD36, a fatty acid transporter, mediates exogenous FA uptake. In one study, terminal differentiation-induced non-coding RNA (*TINCR*) overexpression suppressed CRC cell proliferation and promoted apoptosis through the CD36/PPAR regulatory pathway [129]. In contrast, direct CD36 knockdown or pharmacological inhibition with sulfosuccinimidyl oleate (SSO) reduced CRC cell proliferation and xenograft tumor growth, while combined treatment with SSO and the FASN inhibitor TVB-3664 produced a synergistic antiproliferative effect [26]. CD36 can therefore exert different effects according to the metabolic state of CRC cells. Restoration of *TINCR*/CD36/PPAR signaling is associated with reduced proliferation and increased apoptosis, whereas CRC cells that rely on exogenous fatty acid uptake, particularly following FASN inhibition, become dependent on CD36 to sustain their growth. Thus, *CD36* expression alone does not determine its biological effect, which is also influenced by PPAR signaling and the relative dependence of CRC cells on endogenous fatty acid synthesis versus exogenous fatty acid uptake.

FATP family members represent another potential class of targets for limiting FA uptake. Genetic silencing of solute carrier family 27 member 1 (*SLC27A1*), which encodes FATP1, reduced the migration and invasion of CRC cells, suggesting that FATP1 may represent a potential therapeutic target [78]. In addition, pharmacological inhibition of FATP2 with Lipofermata suppressed colony and sphere formation in colon cancer cells and reduced palmitic acid-driven HCT116 xenograft growth [130].

FABPs are involved in FA transport and intracellular delivery in tumor cells. The FABP4 inhibitor BMS309403, either alone or in combination with cetuximab, significantly inhibits tumor growth in a drug-resistant CRC model [121]. An animal study suggested that pharmacological inhibition of FABP5 with SBFI-26 may reduce stearic acid-induced epithelial proliferation and intestinal tumorigenesis [131]. Collectively, these findings support further investigation of FA uptake as a potential therapeutic target in CRC, particularly in tumors that rely on exogenous lipid supply for growth and treatment adaptation.

### 5.2. Targeting Fatty Acid Synthesis

FA synthesis is an essential metabolic pathway for tumor cells that provides energy and maintains membrane integrity. FASN helps tumor cells grow and survive by increasing FA and LD formation. Orlistat, a known FASN inhibitor, reduces intracellular FA levels by inhibiting the FA synthesis pathway, leading to energy metabolism disruption and subsequent induction of apoptosis. When combined with chemotherapy, orlistat increases cancer cell sensitivity to 5-FU [132].

Epigallocatechin gallate (EGCG), a plant-derived compound, suppresses FASN expression in CRC cells, reduces FA synthesis and ATP production, and thereby induces mitochondrial dysfunction and apoptosis [38]. Additionally, EGCG can increase susceptibility to 5-FU and 2 Gy irradiation [133,134]. The first-in-class FASN inhibitor TVB-2640 has been evaluated in a Phase I trial in patients with advanced solid tumors (NCT02223247) and in a Phase I window trial involving patients with resectable colon or other cancers (NCT02980029) [135,136]. Cluster of differentiation 44 (CD44) is a transmembrane glycoprotein and cell adhesion receptor that interacts with hyaluronan and other extracellular matrix components to regulate cell adhesion, migration, invasion, and tumor progression [153,154]. Pharmacological inhibition of FASN with C75 reduces CD44 expression and attenuates the activation of CD44-associated MET/AKT and FAK/paxillin signaling. These changes promote actin cytoskeletal reorganization and impair CRC cell adhesion, migration, and invasion. More broadly, attenuation of *de novo* lipogenesis inhibits the establishment of hepatic and secondary metastases in vivo [40]. *DNAJC3-AS1*, a lncRNA, controls FASN expression through the EGFR/PI3K/AKT/NF-κB/SREBP1 signaling pathway. *DNAJC3-AS1* knockdown dramatically decreases CRC cell proliferation, migration, and FA accumulation, indicating its potential as a target in metabolic reprogramming [137].

ACLY is the first rate-limiting enzyme in endogenous FA synthesis. Elevated ACLY expression increases resistance to SN-38, the active metabolite of the chemotherapeutic agent irinotecan, whereas ACLY inhibition enhances the sensitivity of CRC cells to SN-38 [138]. Zinc finger DHHC-type containing 6 (ZDHHC6) stabilizes PPARγ through palmitoylation, thereby activating lipid metabolism-related genes such as ACLY. This promotes FA synthesis, intracellular lipid accumulation, and tumor growth. Inhibiting ZDHHC6 expression can considerably delay CRC tumor growth and suppress FAM reprogramming [71]. Firsocostat, an ACC inhibitor, enhanced *CRCMSL*-mediated suppression of CRC growth by reducing membrane fluidity and promoting ferroptosis in preclinical models [35].

SCD1 is essential for cancer cell metabolism, specifically FA synthesis and LD formation. A939572, an SCD1 inhibitor, stimulates FA degradation and oxidation via AMPK activation [54]. MF-438, another SCD1 inhibitor, shows greater antiproliferative activity when combined with 5-FU than when used alone [139]. Moreover, by inhibiting SCD, PluriSln1 counteracts the increased membrane fluidity associated with enhanced ACC activity, thereby reducing CRC cell migration, invasion, and tumor spread [35]. Trichothecin inhibits CRC migration and invasion by suppressing SCD1 and reducing monounsaturated FA levels [41].

SREBPs, key transcription factors involved in FAM, represent important therapeutic targets. SREBP1-mediated lipogenesis promotes lipid droplet accumulation and chemoresistance in CRC, suggesting that this pathway may represent a potential therapeutic target [64,66]. Berberine, a bioactive compound derived from traditional Chinese medicine, inhibits SREBP1 activation, reduces the expression of key lipogenic enzymes, suppresses lipid synthesis, and inhibits CRC xenograft growth [140]. RA-XII, a natural cyclopeptide, efficiently lowers FA levels by blocking SREBP1 expression and *de novo* fatty acid synthesis, consequently preventing tumor development and metastasis [141]. Overall, inhibition of lipogenic pathways represents a promising strategy to disrupt membrane synthesis, energy balance, and metabolic plasticity in CRC, although most current evidence remains preclinical.

### 5.3. Targeting Fatty Acid Oxidation

In specific metabolic contexts, CRC cells can rely on FAO to sustain energy production, suggesting that inhibition of the FAO pathway may represent a promising therapeutic strategy. 2,6-Dihydroxypeperomin B (DHP-B) inhibits FAO by binding to CPT1A and disrupting the interaction between CPT1A and voltage-dependent anion channel 1 (VDAC1), resulting in increased mitochondrial membrane permeability, decreased cellular energy metabolism, and tumor cell death [13]. Valosin-containing protein (VCP) interacts with histone deacetylase 1 (HDAC1) in CRC, increasing its degradation and activating CPT1A transcription, which accelerates FAO. Inhibiting VCP expression can reduce FAO and cell proliferation in CRC. Combining a VCP inhibitor with metformin effectively suppresses tumor development, outperforming either therapy alone [142].

Sirtuin 1 (SIRT1), a deacetylase, alters metabolic pathways in CRC cells by increasing FAO while limiting glycolysis. SIRT1 inhibits glycolysis by deacetylating β-catenin and activates the FAO pathway to meet energy requirements. Targeting SIRT1 with inhibitors such as EX527 markedly reduces tumor growth in vivo, highlighting its promise as a metabolic therapeutic target [143].

VSIG4, a macrophage-associated immune checkpoint molecule, activates JAK2/STAT3 signaling to upregulate PPARγ and enhance FAO in CRC-associated macrophages, thereby promoting M2 polarization. These VSIG4-expressing M2 macrophages facilitate CRC cell proliferation and immune evasion, whereas VSIG4 inhibition enhances the therapeutic efficacy of anti-PD-1 treatment in preclinical CRC models [125,144]. These observations support FAO inhibition as a context-dependent therapeutic approach in CRC, particularly under metabolic stress conditions, but patient selection and metabolic heterogeneity will need to be carefully considered.

### 5.4. Targeting Lipid Droplet Metabolism

As noted above, the hypoxic microenvironment promotes LD accumulation, making LD metabolism a potential therapeutic target [16]. In CRC, LD accumulation is linked to tumor growth, metastasis, and therapeutic resistance. Beyond their role in cancer cell lipid metabolism, LDs can sequester damaged and excess lipids generated during chemotherapy, thereby limiting lipotoxic injury and promoting tumor cell survival [90]. LPCAT2 is an important enzyme in LD biogenesis [50,51]. The LPCAT2 inhibitor TSI-01 markedly reduces LD formation in CRC cells while increasing their sensitivity to chemotherapeutic agents such as 5-FU and oxaliplatin. Furthermore, suppressing LPCAT2 expression or activity could improve chemotherapeutic efficacy by reducing LD formation [51].

GPAT3 promotes resistance to chemotherapeutic agents such as oxaliplatin in CRC by increasing LD formation, and this effect is regulated by deacetylation. Blocking GPAT3 acetylation or utilizing similar inhibitors (for example, AcGPAT3 inhibitors) can drastically reduce LPA/triglyceride production and overcome chemoresistance [50].

High FABP4 expression in CRC cells enhances the production of LDs. Inhibiting FABP4 expression can limit tumor cell motility and LD accumulation, making CRC cells more sensitive to chemotherapeutic medicines such as oxaliplatin. TRIM3 ubiquitinates and degrades FABP4, reducing its expression in CRC cells and thereby decreasing LD production and tumor cell motility [56]. Targeting LD metabolism may therefore help overcome stress adaptation and chemoresistance in CRC, especially when combined with conventional anticancer therapies.

### 5.5. Targeting Hypoxia-Associated Fatty Acid Metabolism

In recent years, targeting hypoxia-associated pathways has attracted increasing interest as a potential therapeutic strategy for CRC. Because the hypoxic TME drives metabolic reprogramming, targeting hypoxia-related pathways represents an important strategy for improving treatment efficacy. One approach is to interfere with HIF-1α-centered signaling and its downstream regulation of lipid storage. Oroxylin A, an active component from the traditional Chinese medicinal plant *Scutellaria baicalensis* Georgi, can inactivate HIF-1α in CRC cells and induce FAM reprogramming characterized by reduced intracellular FA levels and enhanced FAO. It also prevents β-catenin nuclear translocation, suppresses Wnt signaling, and induces G2/M-phase cell-cycle arrest [104]. Exogenous supplementation with the gut microbial metabolite kynurenic acid (KynA) may influence FAM and LD accumulation by controlling HILPDA via the HIF-1α pathway, potentially interfering with CRC growth and metastasis [146]. However, its role in hypoxia requires further investigation.

A complementary strategy is to target the downstream lipid metabolic effectors that sustain CRC adaptation to hypoxia. Ilexgenin A (IA), the major bioactive ingredient from *Ilex hainanensis* Merr., reduces lipid formation in CRC cells under hypoxic conditions by decreasing SREBP1 expression and activation and regulating FASN and ACC [145]. GDF15 expression is positively correlated with HIF-1α expression. Inhibiting GDF15 expression can significantly reduce FAO in tumor cells, thereby suppressing tumor growth and metastasis, revealing the potential of GDF15 as a regulator of FAO [101]. The CPT1 inhibitor etomoxir markedly suppressed glycogen formation in CRC cells under hypoxic conditions, suggesting that FAO is an essential energy source for hypoxic metabolic adaptation. FAO activity appears to be important for hypoxia-induced tumor invasiveness and metastatic capacity, making it a promising therapeutic target beyond HIF-1α or glycolysis inhibition alone. This research suggests that intervening in the still-functioning mitochondrial FAO pathway in hypoxic tumor cells may more effectively reduce their survival advantage and metastatic potential within the hypoxic microenvironment, providing an experimental foundation for developing metabolic therapies targeting hypoxia adaptation in CRC [112]. Together, these studies suggest that targeting either HIF-centered signaling or its downstream lipid metabolic effectors may provide therapeutic opportunities for CRC. These findings highlight the therapeutic potential of targeting hypoxia-associated lipid metabolic adaptation, particularly in CRC cells that retain metabolic flexibility under low-oxygen stress.

### 5.6. Combination Strategies Targeting Fatty Acid Metabolism and Hypoxia-Associated Rsistance

As discussed above, inhibition of fatty acid synthesis and lipid droplet formation can enhance the sensitivity of CRC cells to 5-FU, SN-38, and oxaliplatin. Beyond these direct chemosensitizing effects, ferroptosis- and microenvironment-based combinations provide additional opportunities to overcome treatment resistance.

Ferroptosis-based strategies may also complement chemotherapy in CRC. In oxaliplatin-resistant CRC models, cyclin-dependent kinase 1 (CDK1) inhibition restored oxaliplatin sensitivity by reactivating ACSL4-mediated lipid peroxidation and ferroptosis [147]. These findings suggest that ferroptosis induction may provide an additional approach to overcoming chemotherapy resistance. Manipulating fatty acid metabolism may enhance immunotherapy by altering tumor susceptibility to ferroptosis. Activated CD8^+^ T cells promote tumor-cell ferroptosis through IFN-γ, whereas lipid metabolic remodeling can further amplify this response and improve the efficacy of programmed death-ligand 1 (PD-L1) blockade [148,149]. Hypoxia may profoundly influence this therapeutic interaction, although its effects are context-dependent. On the one hand, HIF-2α activation increases cellular iron accumulation and lipid oxidative stress, thereby creating a ferroptosis-prone state in CRC cells [105]. On the other hand, persistent activation of hypoxia signaling can promote ferroptosis resistance. In RSL3-resistant microsatellite-stable (MSS) CRC cells, increased nuclear HIF-1α directly upregulates prolyl 4-hydroxylase subunit alpha 1 (*P4HA1*) transcription, reduces lipid peroxidation, and maintains resistance to ferroptotic cell death [155]. Accordingly, pharmacological inhibition of HIF-1α restored RSL3 sensitivity. In CT26 models, combining RSL3 with HIF-1α inhibition suppressed subcutaneous tumor growth and liver metastasis, increased the infiltration of CD8^+^ T cells and CD86^+^ macrophages, reduced M2-like macrophage features, and further enhanced the response to anti-PD-1 treatment [155]. Other lipid-related resistance pathways also support this combination strategy. Cytochrome P450 family 1 subfamily B member 1 (CYP1B1)-mediated ACSL4 degradation and ubiquitin-specific peptidase 8 (USP8)-mediated glutathione peroxidase 4 (GPX4) stabilization reduce ferroptosis sensitivity, whereas targeting these pathways improves responses to anti-PD-1 therapy [150,156]. Consistently, RSL3 combined with anti-PD-1 showed synergistic activity in MC38 tumors, while ferroptosis induction together with myeloid-derived suppressor cell blockade sensitized both primary tumors and liver metastases to immune checkpoint blockade [157,158]. Collectively, these findings support combining ferroptosis induction with interventions targeting hypoxia-driven resistance and lipid metabolic adaptation.

Anti-angiogenic therapy, particularly bevacizumab-containing regimens, is an established component of treatment for metastatic CRC and has also provided a clinical platform for combination with immune checkpoint inhibitors [159]. In the AtezoTRIBE trial, adding atezolizumab to 5-FU, leucovorin, oxaliplatin, and irinotecan (FOLFOXIRI) plus bevacizumab prolonged median progression-free survival from 11.5 to 13.1 months, although the updated analysis showed only a modest overall-survival difference in the proficient mismatch repair (pMMR) population and suggested greater benefit in selected tumor mutational burden (TMB)-high or immunoscore IC-high subgroups [160,161]. The effects of angiogenesis inhibition on tumor oxygenation are nevertheless context-dependent: transient vascular normalization may improve perfusion and treatment delivery, whereas sustained vascular depletion can intensify hypoxia and nutrient restriction. Iwamoto et al. showed that anti-vascular endothelial growth factor (VEGF)-induced hypoxia and glucose limitation increased adipose lipolysis, free fatty acid uptake, and CPT1-dependent fatty acid oxidation, allowing CRC tumors to maintain proliferation despite marked vascular depletion; genetic or pharmacological inhibition of CPT1 restored sensitivity to angiogenesis blockade [151]. Consistently, Huang et al. found that pharmacological inhibition of fatty acid oxidation enhanced the efficacy of anti-VEGFA therapy, promoted vascular normalization, and alleviated tissue hypoxia in lipid-rich colorectal liver metastases [152]. These findings suggest that hypoxia-driven lipid metabolic adaptation may contribute to the heterogeneous efficacy and eventual resistance observed with anti-angiogenic therapy, with or without immunotherapy, and provide a mechanistic rationale for further targeting fatty acid metabolism or hypoxia-associated signaling.

## 6. Translational Models and Fatty Acid Metabolic Heterogeneity

### 6.1. Experimental Models for Hypoxia-Associated Fatty Acid Metabolism in CRC

Conventional two-dimensional cultures exposed to defined oxygen tensions remain useful for controlling oxygen and lipid availability and for dissecting individual pathways through genetic or pharmacological interventions. However, these systems cannot reproduce the spatial metabolic heterogeneity observed in three-dimensional CRC models. Spheroids and organoid-based co-culture systems can partially address this limitation by revealing region-specific metabolic states and selected tumor–stromal interactions, although no single model fully reproduces the hypoxic TME [162,163,164,165]. The main features, applications, and limitations of these experimental models are summarized in Table 5.

Multicellular tumor spheroids are among the most established models for examining spatial oxygen and nutrient restriction. Multicellular tumor spheroids generate spatially distinct metabolic compartments that are not apparent in monolayer cultures [163,164]. Spheroids can also reveal lipid heterogeneity that is not apparent in monolayer cultures. Lipidomic analysis of HCT116 spheroids identified distinct profiles among the outer, middle, and core regions, including increased lipid droplet-associated lipid subclasses and polyunsaturated fatty acid-containing lipids in the inner regions [164]. More recently, Głowacka et al. used pimonidazole and a hypoxia-responsive reporter in models including HCT116 and HT29 spheroids to demonstrate that acid-exposed and hypoxic compartments do not completely overlap. The two compartments showed interdependence in their access to unsaturated fatty acids, while SCD1 inhibition also altered the viability of CRC spheroids and patient-derived organoids [163]. These findings illustrate the value of three-dimensional models for resolving spatial FAM adaptations, while also showing that acidity, nutrient restriction, and hypoxia should not be treated as interchangeable conditions. 

Patient-derived organoids preserve important patient-specific tumor characteristics and have been applied to drug-response testing [166]. They may therefore help determine whether hypoxia modifies individual responses to inhibitors of FA uptake, synthesis, desaturation, or oxidation. Nevertheless, conventional patient-derived organoids (PDOs) predominantly represent the epithelial compartment and generally lack functional vasculature and the full complement of immune and stromal cells present in the original tumor [162,167]. Moreover, an internal organoid compartment should not automatically be considered hypoxic unless oxygen levels or validated hypoxia markers are directly assessed. Selected microenvironmental components can be introduced through co-culture. Patient-derived fibroblasts increase tumor-cell heterogeneity in CRC organoid models, whereas adipocytes have been shown to transfer free fatty acids to colon cancer cells, increasing lipid droplet accumulation and CPT1-sensitive FAO [102,162]. The latter study directly supports adipocyte–CRC metabolic coupling, but did not evaluate whether this process is altered by hypoxia. Together, these complementary models support translational investigation of hypoxia-associated FAM in CRC.

### 6.2. Fatty Acid Metabolic Heterogeneity Between Primary CRC and Liver Metastases

Lipid metabolic heterogeneity has been observed between primary CRC and liver metastases. Spatial metabolomic profiling showed that liver metastases contained higher levels of triglycerides, diglycerides, cholesteryl esters, and acylcarnitines than primary tumors, suggesting alterations in lipid synthesis and mitochondrial fatty acid metabolism [168]. In paired clinical samples, *CYP1B1* expression was higher in liver metastases than in primary tumors. *CYP1B1* increased cellular free fatty acids and promoted metastatic CRC cell growth, whereas inhibition of fatty acid synthesis reduced this effect; supplementation with C20:1 or C20:3 partially restored growth after *CYP1B1* knockdown [169].

Heterogeneity also exists among liver metastases. Proteomic analysis of 152 colorectal liver metastases identified splice-driven, complement-associated, and oxidative-metabolic subgroups. The oxidative-metabolic subgroup was enriched in proteins involved in fatty acid metabolism and β-oxidation and was associated with poorer overall survival [170]. Paired primary and liver metastasis-derived organoids have additionally revealed lesion-specific molecular and therapeutic differences, providing a potential platform for investigating fatty acid metabolic variation under controlled oxygen and nutrient conditions [171]. Future studies combining paired primary tumors and liver metastases with spatial profiling, lipidomics, and metabolic tracing may further define the relationship between hypoxia and FAM heterogeneity during CRC liver metastasis.

## 7. Conclusions and Perspectives

FAM reprogramming is now recognized as an important part of CRC progression, especially in the hypoxic TME. Beyond supplying energy and biosynthetic substrates, altered lipid metabolism gives CRC cells greater flexibility to survive and grow under stress. In this setting, hypoxia is not simply a by-product of rapid tumor expansion. It also reshapes how tumor cells acquire, synthesize, oxidize, and store FAs.

One point that emerges clearly from current studies is that the relationship between hypoxia and FAM is not straightforward. Different branches of lipid metabolism do not respond in the same way, and their regulation seems to vary with cellular context, nutrient conditions, and tumor state. This is particularly evident for FAO, whose role in hypoxic CRC cannot be reduced to a simple model of either activation or suppression. A similar shift in view applies to LDs, which are better understood as active participants in metabolic adaptation and treatment response than as passive storage structures.

At the same time, the field is still far from complete. Much of the available evidence remains centered on individual enzymes or pathways, while the broader organization of hypoxia-driven lipid metabolic remodeling in CRC is less well defined. Stromal and immune cells also participate in this remodeling by regulating lipid availability, utilization, and intercellular transfer within the TME. Hypoxia further modifies these interactions, particularly through macrophage lipid remodeling, while its effects on lipid exchange involving adipocytes and CAFs remain less well characterized. The biological context in which these metabolic targets are engaged may therefore be important for their clinical translation.

Looking ahead, a more useful framework will likely come from studying FAM in its spatial, microenvironmental, and disease-site context rather than as a set of isolated pathways. Clarifying where hypoxia-associated lipid programs are engaged, how they vary between primary tumors and liver metastases, and how they influence responses to existing therapies should be important next steps. In practice, the clinical value of targeting FAM may lie in rational combinations with chemotherapy, ferroptosis induction, immunotherapy, or anti-angiogenic therapy rather than in isolated pathway inhibition. Experimental models that reproduce spatial hypoxia, patient-specific tumor characteristics, and selected microenvironmental interactions will be important for evaluating these strategies. Integrating such models with spatial profiling, lipidomics, and hypoxia-related biomarkers may support more precise evaluation of FAM-associated vulnerabilities in CRC.

Overall, the hypoxia–FAM axis offers a useful framework for understanding how CRC cells and surrounding stromal and immune populations adapt to metabolic stress and sustain malignant behavior. A clearer understanding of this process should help refine current models of metabolic reprogramming and may also support the development of more effective therapeutic strategies in CRC.

## Figures and Tables

**Table 1:** Key regulators of fatty acid metabolism reprogramming in colorectal cancer.

Pathway	Molecules	Expression	Feature/Regulatory Mechanism	Biological Relevance in CRC	Reference
Fatty acid uptake	FABPs	Upregulated	Promotes LD formation via AKT pathway; Undergoes DNA demethylation and NF-κB pathway activation	Poor prognosis; Tumor invasion and metastasis; Cell-cycle regulation; Higher FABP4 expression in stage III–IV CRC	[20,21,22,23,24]
	FAT/CD36	Context-dependent	Mediates FA uptake and ATP production; Regulates MMP28; Inhibits downstream aerobic glycolysis	Poor prognosis; Promotes tumor growth, invasion and metastasis	[25,26,27,28,29,30]
	FATPs	Upregulated	*FATP5* knockdown increases proliferation and reduces G2/M-phase accumulation	Correlated with prognosis	[31]
*De novo* synthesis	ACLY	Upregulated	Interacts with β-catenin; Activated by HOXA13	Promotes tumor proliferation, metastasis and therapeutic resistance	[32,33]
	ACC	Upregulated	Regulated by AMPK, *CRCMSL* and REG4; Promotes membrane fluidity	Regulates tumor growth, metastasis and apoptosis	[34,35,36]
	FASN	Upregulated	Converts acetyl-CoA and malonyl-CoA to FAs; Increases ATP production; Activates β-catenin signaling	Increases stem-like properties; Supports tumor growth and metastasis	[37,38,39,40]
	SCD	Upregulated	Converts SFAs to MUFAs; Promotes EMT and ATP production	Enhances tumor invasion and metastasis	[41,42,43]
Fatty acid oxidation	CPT1A	Upregulated	Enhances FAO and suppresses anoikis	Promotes metastatic potential	[44]
	CPT1C	Upregulated	Increases FAO activity	Promotes proliferation, migration, and cell-cycle progression	[45]
	CPT2	Downregulated	Associated with p53 regulation	Influences progression, stemness, and chemoresistance	[46,47]
	ACOX1	Context-dependent	Regulated by *SLC9A5*	Associates with tumor proliferation and metastasis	[48,49]
Storage & Lipolysis	GPAT3	Upregulated	Catalyzes LPA synthesis; Promotes LD formation	Enhances therapeutic resistance and tumor proliferation	[50]
	LPCAT2	Upregulated	Supports phospholipid synthesis in LDs; Buffers lipotoxicity	Promotes therapeutic resistance	[51]
	SCD1	Upregulated	Promotes LD accumulation; Inhibition enhances CD8^+^ T cells activation and IFN-γ production	Contributes to therapeutic resistance and immune escape	[52,53,54]
	FABP6	Upregulated	Transcriptionally activated by KLF5	Promotes therapeutic resistance and tumor proliferation	[55]
	FABP4	Upregulated	Degraded by TRIM3 via ubiquitination	Promotes therapeutic resistance and tumor invasion	[56]
	ATGL	Upregulated	Degrades triglycerides; Mediated by obesity	Promotes tumor proliferation	[57,58]
	HILPDA	Upregulated	Inhibits ATGL-mediated intracellular lipolysis; Buffers lipotoxicity	Promotes tumor proliferation	[59,60]
	PLIN2	Upregulated	Promotes CD36-mediated EMT; Promotes lipid droplet homeostasis	Potential biomarker for early-stage CRC; Promotes EMT and tumor progression	[61,62]
Regulation	SREBP	Upregulated	Master transcription factor for FASN, SCD, ACC1; Enhanced by PTMA and *ZFAS1*	Promotes tumor proliferation, metastasis, and therapeutic resistance	[63,64,65,66]
	PPARα	Context-dependent	Upregulated by PTPRO to enhance FAO; Downregulated by AKT/mTOR	Context-dependent effects on tumor growth, invasion, and treatment response	[67,68,69]
	PPARβ/δ	Upregulated	Activates CPT1A in intestinal stem cells and enhances FAO	Promotes tumor proliferation	[70]
	PPARγ	Downregulated	Stabilized by ZDHHC6-mediated palmitoylation; Activates ACLY	Promotes tumor proliferation	[69,71]
	REG4	Context-dependent	Represses ACC1 and ACLY while affecting LD synthesis and assembly	Promotes LD accumulation and chemoresistance	[36]
	KLF5	Upregulated	Transcriptionally activates FABP6	Promotes therapeutic resistance and tumor proliferation	[55]

Abbreviations: acetyl-CoA, acetyl coenzyme A; ACC, acetyl-CoA carboxylase; ACC1, acetyl-CoA carboxylase 1; ACLY, ATP-citrate lyase; ACOX1, acyl-CoA oxidase 1; AKT, protein kinase B; AMPK, AMP-activated protein kinase; ATGL, adipose triglyceride lipase; ATP, adenosine triphosphate; CPT1A, carnitine palmitoyltransferase 1A; CPT1C, carnitine palmitoyltransferase 1C; CPT2, carnitine palmitoyltransferase 2; CRC, colorectal cancer; *CRCMSL*, colorectal cancer metastasis-suppressed long non-coding RNA; EMT, epithelial–mesenchymal transition; FA, fatty acid; FABP, fatty acid-binding protein; FAO, fatty acid oxidation; FASN, fatty acid synthase; FAT/CD36, fatty acid translocase/cluster of differentiation 36; FATP, fatty acid transport protein; GPAT3, glycerol-3-phosphate acyltransferase 3; HILPDA, hypoxia-inducible lipid droplet-associated protein; HOXA13, homeobox A13; IFN-γ, interferon gamma; KLF5, Krüppel-like factor 5; LD, lipid droplet; LPA, lysophosphatidic acid; LPCAT2, lysophosphatidylcholine acyltransferase 2; MMP28, matrix metalloproteinase 28; malonyl-CoA, malonyl coenzyme A; mTOR, mechanistic target of rapamycin; MUFA, monounsaturated fatty acid; NF-κB, nuclear factor kappa B; PLIN2, perilipin 2; PPAR, peroxisome proliferator-activated receptor; PTMA, prothymosin α; PTPRO, protein tyrosine phosphatase receptor type O; REG4, regenerating gene 4; SCD, stearoyl-CoA desaturase; SCD1, stearoyl-CoA desaturase 1; SFA, saturated fatty acid; SLC9A5, solute carrier family 9 member A5; SREBP, sterol regulatory element-binding protein; TRIM3, tripartite motif-containing 3; ZDHHC6, zinc finger DHHC-type containing 6; *ZFAS1*, zinc finger antisense 1.

**Table 2:** Key regulators of hypoxia-associated fatty acid metabolism in colorectal cancer.

Regulator	Target Molecule	Expression	Biological Relevance in CRC	Remark	Reference
HIF-1α	FASN	Upregulated	Enhances lipid uptake, endogenous lipogenesis, and lipid storage; Suppresses selected FAO pathways; Supports tumor growth and cell-cycle progression	NA	[104]
	PLIN2	Upregulated			
	FABPs	Upregulated			
	CPT1	Downregulated			
HIF-1α	CPT1C	Upregulated	Increases FAO rate; Promotes proliferation, migration, and cell-cycle progression; Correlates with poorer relapse-free survival	NA	[45]
HIF-1α	HILPDA	Upregulated	Increases LD accumulation; Buffers lipotoxicity and supports tumor proliferation and metastasis	NA	[60]
GDF15	CPT1	Upregulated	Enhances FAO; Supports CRC cell growth and migration	Regulated by HIF-1α	[101]
	CPT2	Upregulated			
	ACSL1	Upregulated			
	CD36	Upregulated			
MED15	CPT1A	Downregulated	Promotes LD accumulation; Supports tumor growth	Regulated by both HIF-1α and HIF-2α	[100]
CD147	PPARα	Downregulated	Increases dependence on glycolysis under hypoxia; Enhances therapeutic resistance	Activates the PI3K/AKT/mTOR pathway to further upregulate HIF-1α	[99]
HIF-2α	HILPDA	Upregulated	Regulates iron metabolism and lipid droplet accumulation; Influences tumor cell growth in hypoxic and oxidative stress conditions	NA	[105]
	PLIN2	Upregulated			
*SFRP2*	SREBP1	Upregulated	Promotes lipid synthesis; Associated with EMT; Contributes to therapeutic resistance and tumor proliferation	NA	[106]
*ITLN1*		Downregulated			
PPARα	ACOX1	Upregulated	Enhances FAO; Supports tumor growth in acidic and hypoxic conditions	NA	[107]

Abbreviations: ACOX1, acyl-CoA oxidase 1; ACSL1, acyl-CoA synthetase long-chain family member 1; AKT, protein kinase B; CD147, cluster of differentiation 147; CD36, cluster of differentiation 36; CPT1, carnitine palmitoyltransferase 1; CPT1A, carnitine palmitoyltransferase 1A; CPT1C, carnitine palmitoyltransferase 1C; CPT2, carnitine palmitoyltransferase 2; CRC, colorectal cancer; EMT, epithelial–mesenchymal transition; FABP, fatty acid-binding protein; FAO, fatty acid oxidation; FASN, fatty acid synthase; GDF15, growth differentiation factor 15; HIF-1α, hypoxia-inducible factor 1α; HIF-2α, hypoxia-inducible factor 2α; HILPDA, hypoxia-inducible lipid droplet-associated protein; *ITLN1*, intelectin 1; LD, lipid droplet; MED15, mediator complex subunit 15; mTOR, mechanistic target of rapamycin; NA, not applicable; PI3K, phosphoinositide 3-kinase; PLIN2, perilipin 2; PPARα, peroxisome proliferator-activated receptor α; *SFRP2*, secreted frizzled-related protein 2; SREBP1, sterol regulatory element-binding protein 1.

**Table 3:** Fatty acid metabolism in stromal and immune cells in colorectal cancer.

Cell Type	Interaction	FAM Alteration	Feature/Regulatory Mechanism	Biological Relevance in CRC	Reference
Adipocytes	Lipid transfer to CRC cells	FFA transfer; FAO upregulation	Autophagy through AMPK activation	Survival under nutrient deprivation	[102]
Adipocytes	Reprogramming by CRC cells	LD redistribution; Reduced lipid storage; Glycolytic shift	Adipocyte dedifferentiation	Stromal state supporting tumor progression	[120]
Adipocytes	Metabolic support of CRC cells	Altered FA handling	FABP4 upregulation	Cetuximab resistance	[121]
CAFs	Lipid transfer to CRC cells	Lipid supply and uptake	FASN; CD36	Migration and metastasis	[29]
CAFs	Lipid transfer to CRC cells	Incorporation of FAs into unsaturated phosphatidylcholines	Membrane lipid remodeling	Peritoneal dissemination	[118]
CAFs	Intrinsic metabolic adaptation	FAO upregulation	CPT1A	CAF survival; Peritoneal metastasis	[119]
CAFs	Reprogramming by CRC cells	Lipid accumulation	KRAS; TFCP2; VEGFA	Angiogenesis and tumor progression	[122]
CAFs	Reprogramming by CRC cells	Acetyl-CoA production through ACLY	ACLY activation; CXCL5	Liver metastatic niche formation	[123]
CAFs	Reprogramming by CRC cells	Palmitic acid accumulation	Cytokine signaling through NF-κB	ECM stiffening	[124]
Macrophages	Adaptation to the CRC microenvironment	LD formation; FAO upregulation	Mitochondrial metabolic remodeling	Immunosuppressive phenotype	[92]
Macrophages	Macrophage metabolic regulation in CRC	FAO upregulation	VSIG4	M2-like polarization; Immune escape	[125]
Macrophages	Adaptation to hypoxia	Reduced lipid biosynthesis	CSF1R inhibition; HIF-2α destabilization; DPD reduction	Increased 5-FU sensitivity	[126]
Macrophages	Lipid transfer under hypoxia	LD formation; FAO; Transfer of exosomes rich in TGs	HIF-1α; FABP7; DGAT1	Liver metastasis; Impaired T cell function	[103]
Neutrophils	Lipid transfer to CRC cells	LD formation; Lipid transfer	IL-33	Metastatic reactivation	[93]
MDSCs and CD8^+^ T cells	Response to dietary FAs	Altered immune cell composition	Palmitic acid; CLA	Increased immunosuppression	[127]
DCs and CD8^+^ T cells	Response to SCD1 inhibition	Reduced FA desaturation	SCD1 inhibition	Enhanced antitumor immunity and anti-PD-1 efficacy	[52]
NK cells	Interaction with hypoxic CRC cells	Altered DDA metabolism	FADS1	Reduced NK cell cytotoxicity	[128]

Abbreviations: 5-FU, 5-fluorouracil; ACLY, ATP-citrate lyase; AMPK, AMP-activated protein kinase; CAF, cancer-associated fibroblast; CD36, cluster of differentiation 36; CLA, conjugated linoleic acid; CPT1A, carnitine palmitoyltransferase 1A; CRC, colorectal cancer; CSF1R, colony-stimulating factor 1 receptor; CXCL5, C-X-C motif chemokine ligand 5; DC, dendritic cell; DDA, docosadienoic acid; DGAT1, diacylglycerol acyltransferase 1; DPD, dihydropyrimidine dehydrogenase; ECM, extracellular matrix; FA, fatty acid; FABP4, fatty acid-binding protein 4; FABP7, fatty acid-binding protein 7; FADS1, fatty acid desaturase 1; FAM, fatty acid metabolism; FAO, fatty acid oxidation; FASN, fatty acid synthase; FFA, free fatty acid; HIF-1α, hypoxia-inducible factor 1α; HIF-2α, hypoxia-inducible factor 2α; IL-33, interleukin 33; KRAS, Kirsten rat sarcoma viral oncogene homolog; LD, lipid droplet; MDSC, myeloid-derived suppressor cell; NF-κB, nuclear factor kappa B; NK, natural killer; PD-1, programmed cell death protein 1; SCD1, stearoyl-CoA desaturase 1; TFCP2, transcription factor CP2; TG, triglyceride; VEGFA, vascular endothelial growth factor A; VSIG4, V-set and immunoglobulin domain-containing 4.

**Table 4:** Representative therapeutic and combination strategies targeting fatty acid metabolism in colorectal cancer.

Pathway	Target	Intervention	Combination	Development Stage	Mechanism and Therapeutic Relevance	Reference
Uptake	CD36	*TINCR* overexpression	–	Preclinical	Restores *TINCR*/CD36/PPAR signaling; Inhibits proliferation and promotes apoptosis	[129]
	CD36	SSO	TVB-3664	Preclinical	CD36 inhibitor; Inhibits CD36-dependent FA uptake; Reduces tumor growth; Enhances FASN inhibition	[26]
	FATP1	*SLC27A1* inhibition	–	Preclinical	Genetic silencing; Reduces tumor migration and invasion	[78]
	FATP2	Lipofermata	2-BP	Preclinical	FATP2 inhibitor; Suppresses colony and sphere formation and xenograft growth	[130]
	FABP4	BMS309403	Cetuximab	Preclinical	FABP4 inhibitor; Inhibits tumor growth in cetuximab-resistant CRC	[121]
	FABP5	SBFI-26	–	Preclinical	FABP5 inhibitor; Reduces intestinal tumorigenesis	[131]
*De novo* synthesis	FASN	Orlistat	5-FU	Preclinical	FASN inhibitor; Reduces FA synthesis; Disrupts energy metabolism; Induces apoptosis	[132]
	FASN	EGCG	5-FU; 2 Gy	Preclinical	Plant polyphenol; Inhibits FASN expression; Reduces ATP synthesis; Induces mitochondrial damage and apoptosis	[38,133,134]
	FASN	TVB-2640	–	Phase I (NCT02223247; NCT02980029)	Highly selective FASN inhibitor; Reduces FA and ATP levels	[135,136]
	FASN	C75	–	Preclinical	FASN inhibitor; Downregulates CD44 expression; Reduces migration and metastatic potential	[40]
	FASN	*DNAJC3-AS1* knockdown	–	Preclinical	lncRNA-targeted intervention; Suppresses FASN expression and FA accumulation	[137]
	ACLY	GSK165	SN-38	Preclinical	ACLY inhibitor; Enhances sensitivity to SN-38	[138]
	ACLY	ZDHHC6 inhibition	–	Preclinical	Reduces ACLY-related lipogenesis and lipid accumulation	[71]
	ACC	Firsocostat	–	Preclinical	ACC inhibitor; Inhibits membrane fluidity; Increases ferroptosis	[35]
	SCD1	A939572	–	Preclinical	SCD1 inhibitor; Promotes FA degradation and oxidation via AMPK pathway	[54]
	SCD1	MF-438	5-FU	Preclinical	SCD1 inhibitor; Reduces FA synthesis	[139]
	SCD1	Trichothecin	–	Preclinical	Sesquiterpenoid compound; Reduces FA synthesis and invasion	[41]
	SREBP1	Berberine	–	Preclinical	Bioactive natural compound; Inhibits SREBP1 activation and lipogenic gene expression	[140]
	SREBP1	RA-XII	–	Preclinical	Natural cyclopeptide; Downregulates expression of FA synthesis genes	[141]
FAO	CPT1	DHP-B	–	Preclinical	Binds and inhibits CPT1A; Increases mitochondrial membrane permeability; Etomoxir was used as a comparator	[13]
	VCP	CB-5083	Metformin	Preclinical	VCP inhibitor; Reduces CPT1A transcription; Suppresses FAO and tumor growth	[142]
	SIRT1	EX527	Etomoxir	Preclinical	SIRT1 inhibitor; Suppresses SIRT1-mediated FAO and reduces tumor growth	[143]
	VSIG4	VSIG4 inhibition	Anti-PD-1	Preclinical	Inhibits VSIG4 expression; Reduces M2 macrophage polarization and FAO	[125,144]
Lipid droplet formation	LPCAT2	TSI-01	5-FU/Oxaliplatin	Preclinical	LPCAT2 inhibitor; Reduces LD accumulation	[51]
	GPAT3	AcGPAT3 inhibitor	Oxaliplatin	Preclinical	Inhibits GPAT3 acetylation; Reduces LPA/TG synthesis	[50]
	FABP4	TRIM3 overexpression	Oxaliplatin	Preclinical	Promotes ubiquitin-mediated degradation of FABP4; Reduces LD accumulation and tumor cell motility	[56]
Hypoxia	HIF-1α	Oroxylin A	–	Preclinical	Natural flavonoid; Inactivates HIF-1α; Reduces FA levels	[104]
	SREBP1/FASN/ACC	Ilexgenin A	–	Preclinical	Natural compound; Inhibits SREBP1 activation and FASN/ACC expression under hypoxia; Reduces lipid accumulation	[145]
	GDF15	GDF15 inhibition	–	Preclinical	Inhibits GDF15 expression; Reduces FAO; Suppresses tumor growth and metastasis	[101]
	CPT1	Etomoxir	–	Preclinical	CPT1 inhibitor; Reverses hypoxia-induced glycogen accumulation; Inhibits FAO-dependent invasion and metastasis	[112]
	HILPDA	KynA	–	Preclinical	Gut microbiota metabolite; Modulates the HIF-1α/HILPDA axis and may reduce LD accumulation; Its role in hypoxia requires further investigation	[146]
Ferroptosis-based therapy	CDK1/ACSL4	RO-3306	Oxaliplatin	Preclinical	CDK1 inhibitor; Reactivates ACSL4-mediated lipid peroxidation and restores oxaliplatin sensitivity	[147]
	ACSL4-mediated PUFA metabolism	Arachidonic acid	Anti-PD-L1	Preclinical	Polyunsaturated fatty acid; Enhances ACSL4-dependent lipid peroxidation and improves the response to PD-L1 blockade	[148,149]
	CYP1B1/ACSL4	*CYP1B1* knockdown	Anti-PD-1	Preclinical	Gene-silencing strategy; Prevents ACSL4 degradation and sensitizes CRC tumors to PD-1 blockade	[150]
Anti-angiogenic therapy	CPT1	Etomoxir	Anti-VEGF antibody	Preclinical	CPT1 inhibitor; Blocks hypoxia-associated FAO adaptation and restores sensitivity to anti-VEGF therapy	[151]
	FAO	Etomoxir	Anti-VEGFA antibody	Preclinical	FAO inhibitor; Enhances anti-VEGFA efficacy, promotes vascular normalization, and alleviates tissue hypoxia in lipid-rich CRLM	[152]

Abbreviations: 2-BP, 2-bromopalmitate; 5-FU, 5-fluorouracil; ACC, acetyl-CoA carboxylase; AcGPAT3, acetylated glycerol-3-phosphate acyltransferase 3; ACLY, ATP-citrate lyase; ACSL4, acyl-CoA synthetase long-chain family member 4; AKT, protein kinase B; AMPK, AMP-activated protein kinase; ATP, adenosine triphosphate; CD36, cluster of differentiation 36; CD44, cluster of differentiation 44; CDK1, cyclin-dependent kinase 1; CPT1, carnitine palmitoyltransferase 1; CPT1A, carnitine palmitoyltransferase 1A; CRC, colorectal cancer; CRLM, colorectal liver metastasis; *CYP1B1*, cytochrome P450 family 1 subfamily B member 1; DHP-B, 2,6-dihydroxypeperomin B; *DNAJC3-AS1*, DNAJC3 antisense RNA 1; EGCG, epigallocatechin gallate; FA, fatty acid; FABP, fatty acid-binding protein; FAO, fatty acid oxidation; FASN, fatty acid synthase; FATP, fatty acid transport protein; GDF15, growth differentiation factor 15; GPAT3, glycerol-3-phosphate acyltransferase 3; HIF-1α, hypoxia-inducible factor 1α; HILPDA, hypoxia-inducible lipid droplet-associated protein; KynA, kynurenic acid; LD, lipid droplet; lncRNA, long non-coding RNA; LPA, lysophosphatidic acid; LPCAT2, lysophosphatidylcholine acyltransferase 2; PD-1, programmed cell death protein 1; PD-L1, programmed death-ligand 1; PPAR, peroxisome proliferator-activated receptor; PUFA, polyunsaturated fatty acid; SCD1, stearoyl-CoA desaturase 1; SIRT1, sirtuin 1; *SLC27A1*, solute carrier family 27 member 1; SN-38, 7-ethyl-10-hydroxycamptothecin; SREBP1, sterol regulatory element-binding protein 1; SSO, sulfosuccinimidyl oleate; TG, triglyceride; *TINCR*, terminal differentiation-induced non-coding RNA; TRIM3, tripartite motif-containing 3; VCP, valosin-containing protein; VEGF, vascular endothelial growth factor; VEGFA, vascular endothelial growth factor A; VSIG4, V-set and immunoglobulin domain-containing 4; ZDHHC6, zinc finger DHHC-type containing 6.

**Table 5:** Experimental models for hypoxia-associated fatty acid metabolism in colorectal cancer.

Model	Representation of Hypoxia and Spatial Features	Applications to FAM Research in CRC	Patient/TME Relevance	Main Limitations	Reference
Two-dimensional hypoxic culture	Provides uniform and experimentally controlled oxygen exposure	Enables mechanistic analysis of fatty acid uptake, synthesis, desaturation, storage, and oxidation under defined oxygen and lipid conditions	Low	Does not reproduce the spatial metabolic heterogeneity or selected tumor–stromal interactions observed in three-dimensional CRC models	[104,112]
Multicellular tumor spheroids	Generate spatially distinct metabolic compartments and region-specific lipid profiles; Hypoxic and acid-exposed compartments may not completely overlap	Reveals region-specific lipid profiles, spatial lipid remodeling, and differential access to unsaturated fatty acids	Moderate; Mainly cell-line-derived	Hypoxia, acidity, nutrient restriction, and waste accumulation occur simultaneously and are difficult to separate	[163,164]
Patient-derived organoids	Internal oxygen gradients may occur but require direct validation using oxygen measurements or established hypoxia markers	Supports patient-specific evaluation of responses to inhibitors of fatty acid uptake, synthesis, desaturation, or oxidation	High for tumor epithelial characteristics	Generally lacks functional vasculature and complete immune, fibroblast, adipocyte, and endothelial components	[162,166,167]
Heterotypic co-culture models	Hypoxia was not evaluated in the cited FAM-related co-culture study	Enables assessment of stromal–tumor metabolic interactions, including adipocyte-derived fatty acid transfer, lipid droplet accumulation, and CPT1-sensitive FAO	Incorporates selected stromal or immune components	Cell composition is incomplete and model-dependent; direct evidence integrating hypoxia, FAM, and microenvironmental interactions remains limited	[102,162]

Abbreviations: CPT1, carnitine palmitoyltransferase 1; CRC, colorectal cancer; FAM, fatty acid metabolism; FAO, fatty acid oxidation; TME, tumor microenvironment.

## Data Availability

No new data were created or analyzed in this study. Data sharing is not applicable to this article.

## Abbreviations

The following abbreviations are used in this manuscript:

Abbreviation	Full Term
5-FU	5-fluorouracil
ACC	Acetyl-CoA carboxylase
acetyl-CoA	Acetyl coenzyme A
ACLY	ATP-citrate lyase
ACOX	Acyl-CoA oxidase
ACOX1	Acyl-CoA oxidase 1
ACSL	Acyl-CoA synthetase long-chain family
ACSS	Acetyl-CoA synthetase
AKT	Protein kinase B
AMPK	AMP-activated protein kinase
ATGL	Adipose triglyceride lipase
ATP	Adenosine triphosphate
CAF	Cancer-associated fibroblast
CD147	Cluster of differentiation 147
CD44	Cluster of differentiation 44
CDK1	Cyclin-dependent kinase 1
CPT1	Carnitine palmitoyltransferase 1
CPT2	Carnitine palmitoyltransferase 2
CRC	Colorectal cancer
*CRCMSL*	Colorectal cancer metastasis-suppressed long non-coding RNA
CXCL5	C-X-C motif chemokine ligand 5
CYP1B1	Cytochrome P450 family 1 subfamily B member 1
DGAT1	Diacylglycerol acyltransferase 1
DHP-B	2,6-dihydroxypeperomin B
*DNAJC3-AS1*	DNAJC3 antisense RNA 1
DPD	Dihydropyrimidine dehydrogenase
EGCG	Epigallocatechin gallate
EGFR	Epidermal growth factor receptor
EMT	Epithelial–mesenchymal transition
FA	Fatty acid
FABP	Fatty acid-binding protein
FADS1	Fatty acid desaturase 1
FAM	Fatty acid metabolism
FAO	Fatty acid oxidation
FASN	Fatty acid synthase
FAT/CD36	Fatty acid translocase/cluster of differentiation 36
FATP	Fatty acid transport protein
FOLFOXIRI	5-fluorouracil, leucovorin, oxaliplatin, and irinotecan
GDF15	Growth differentiation factor 15
*GLUT1*	Glucose transporter 1
GPAT3	Glycerol-3-phosphate acyltransferase 3
GPX4	Glutathione peroxidase 4
HDAC1	Histone deacetylase 1
HIF	Hypoxia-inducible factor
HIF-1α	Hypoxia-inducible factor 1α
HIF-2α	Hypoxia-inducible factor 2α
HILPDA	Hypoxia-inducible lipid droplet-associated protein
*HMOX1*	Heme oxygenase 1
HOXA13	Homeobox A13
IA	Ilexgenin A
IFN-γ	Interferon gamma
IGF-1R	Insulin-like growth factor 1 receptor
ISR	Integrated stress response
*ITLN1*	Intelectin 1
KLF5	Krüppel-like factor 5
KRAS	Kirsten rat sarcoma viral oncogene homolog
KynA	Kynurenic acid
LD	Lipid droplet
LPA	Lysophosphatidic acid
LPCAT2	Lysophosphatidylcholine acyltransferase 2
MED15	Mediator complex subunit 15
MMP28	Matrix metalloproteinase 28
MSS	Microsatellite-stable
mTOR	Mechanistic target of rapamycin
MUFA	Monounsaturated fatty acid
NADPH	Reduced nicotinamide adenine dinucleotide phosphate
NF-κB	Nuclear factor kappa B
NK	Natural killer
*P4HA1*	Prolyl 4-hydroxylase subunit alpha 1
PD-1	Programmed cell death protein 1
PD-L1	Programmed death-ligand 1
PDO	Patient-derived organoid
PI3K	Phosphoinositide 3-kinase
PLIN2	Perilipin 2
pMMR	Proficient mismatch repair
PPAR	Peroxisome proliferator-activated receptor
PTMA	Prothymosin α
PUFA	Polyunsaturated fatty acid
REG4	Regenerating gene 4
ROS	Reactive oxygen species
SCD	Stearoyl-CoA desaturase
SFA	Saturated fatty acid
*SFRP2*	Secreted frizzled-related protein 2
SIRT1	Sirtuin 1
*SLC27A1*	Solute carrier family 27 member 1
SREBP	Sterol regulatory element-binding protein
SSO	Sulfosuccinimidyl oleate
TCA	Tricarboxylic acid cycle
TFCP2	Transcription factor CP2
*TINCR*	Terminal differentiation-induced non-coding RNA
TMB	Tumor mutational burden
TME	Tumor microenvironment
UFA	Unsaturated fatty acid
UPR	Unfolded protein response
USP8	Ubiquitin-specific peptidase 8
VCP	Valosin-containing protein
VDAC1	Voltage-dependent anion channel 1
VEGF	Vascular endothelial growth factor
VEGFA	Vascular endothelial growth factor A
VSIG4	V-set and immunoglobulin domain-containing 4
ZDHHC6	Zinc finger DHHC-type containing 6
*ZFAS1*	Zinc finger antisense 1

## References

[1] Abedizadeh R., Majidi F., Khorasani H.R., Abedi H., Sabour D. (2024). Colorectal cancer: A comprehensive review of carcinogenesis, diagnosis, and novel strategies for classified treatments. Cancer Metastasis Rev..

[2] Sung H., Filho A.M., Laversanne M., Ferlay J., Siegel R.L., Soerjomataram I., Jemal A., Bray F. (2026). Global cancer statistics 2024: GLOBOCAN estimates of incidence and mortality worldwide for 34 cancers in 186 countries. CA Cancer J. Clin..

[3] Pan H., Zhao Z., Deng Y., Zheng Z., Huang Y., Huang S., Chi P. (2022). The global, regional, and national early-onset colorectal cancer burden and trends from 1990 to 2019: Results from the Global Burden of Disease Study 2019. BMC Public Health.

[4] Spaander M.C.W., Zauber A.G., Syngal S., Blaser M.J., Sung J.J., You Y.N., Kuipers E.J. (2023). Young-onset colorectal cancer. Nat. Rev. Dis. Primers.

[5] Choi J., Jia G., Wen W., Shu X.O., Zheng W. (2021). Healthy lifestyles, genetic modifiers, and colorectal cancer risk: A prospective cohort study in the UK Biobank. Am. J. Clin. Nutr..

[6] Huyghe J.R., Bien S.A., Harrison T.A., Kang H.M., Chen S., Schmit S.L., Conti D.V., Qu C., Jeon J., Edlund C.K. (2019). Discovery of common and rare genetic risk variants for colorectal cancer. Nat. Genet..

[7] Legge D.N., Collard T.J., Stanko E., Hoskin A.J., Holt A.K., Bull C.J., Kollareddy M., Bellamy J., Groves S., Ma E.H. (2024). Identifying targetable metabolic dependencies across colorectal cancer progression. Mol. Metab..

[8] Satoh K., Yachida S., Sugimoto M., Oshima M., Nakagawa T., Akamoto S., Tabata S., Saitoh K., Kato K., Sato S. (2017). Global metabolic reprogramming of colorectal cancer occurs at adenoma stage and is induced by MYC. Proc. Natl. Acad. Sci. USA.

[9] Seo J.Y., Jin E.H., Chung G.E., Kim Y.S., Bae J.H., Yim J.Y., Han K.D., Yang S.Y. (2023). The risk of colorectal cancer according to obesity status at four-year intervals: A nationwide population-based cohort study. Sci. Rep..

[10] Nieman K.M., Kenny H.A., Penicka C.V., Ladanyi A., Buell-Gutbrod R., Zillhardt M.R., Romero I.L., Carey M.S., Mills G.B., Hotamisligil G.S. (2011). Adipocytes promote ovarian cancer metastasis and provide energy for rapid tumor growth. Nat. Med..

[11] Yao C.H., Fowle-Grider R., Mahieu N.G., Liu G.Y., Chen Y.J., Wang R., Singh M., Potter G.S., Gross R.W., Schaefer J. (2016). Exogenous Fatty Acids Are the Preferred Source of Membrane Lipids in Proliferating Fibroblasts. Cell Chem. Biol..

[12] Cui H., Wang Y., Zhou T., Qu L., Zhang X., Wang Y., Han M., Yang S., Ren X., Wang G. (2024). Targeting DGAT1 inhibits prostate cancer cells growth by inducing autophagy flux blockage via oxidative stress. Oncogene.

[13] Hu A., Wang H., Xu Q., Pan Y., Jiang Z., Li S., Qu Y., Hu Y., Wu H., Wang X. (2023). A novel CPT1A covalent inhibitor modulates fatty acid oxidation and CPT1A-VDAC1 axis with therapeutic potential for colorectal cancer. Redox Biol..

[14] Li Z., Ji B.W., Dixit P.D., Tchourine K., Lien E.C., Hosios A.M., Abbott K.L., Rutter J.C., Westermark A.M., Gorodetsky E.F. (2022). Cancer cells depend on environmental lipids for proliferation when electron acceptors are limited. Nat. Metab..

[15] Terry A.R., Nogueira V., Rho H., Ramakrishnan G., Li J., Kang S., Pathmasiri K.C., Bhat S.A., Jiang L., Kuchay S. (2023). CD36 maintains lipid homeostasis via selective uptake of monounsaturated fatty acids during matrix detachment and tumor progression. Cell Metab..

[16] Bensaad K., Favaro E., Lewis C.A., Peck B., Lord S., Collins J.M., Pinnick K.E., Wigfield S., Buffa F.M., Li J.L. (2014). Fatty acid uptake and lipid storage induced by HIF-1α contribute to cell growth and survival after hypoxia-reoxygenation. Cell Rep..

[17] Harada A., Yasumizu Y., Harada T., Fumoto K., Sato A., Maehara N., Sada R., Matsumoto S., Nishina T., Takeda K. (2025). Hypoxia-induced Wnt5a-secreting fibroblasts promote colon cancer progression. Nat. Commun..

[18] Kalish B.T., Fallon E.M., Puder M. (2012). A tutorial on fatty acid biology. JPEN J. Parenter. Enter. Nutr..

[19] Singh K., Bunzel G., Graf B., Yip K.M., Neumann-Schaal M., Stark H., Chari A. (2023). Reconstruction of a fatty acid synthesis cycle from acyl carrier protein and cofactor structural snapshots. Cell.

[20] Kawaguchi K., Senga S., Kubota C., Kawamura Y., Ke Y., Fujii H. (2016). High expression of Fatty Acid-Binding Protein 5 promotes cell growth and metastatic potential of colorectal cancer cells. FEBS Open Bio.

[21] Prayugo F.B., Kao T.J., Anuraga G., Ta H.D.K., Chuang J.Y., Lin L.C., Wu Y.F., Wang C.Y., Lee K.H. (2021). Expression Profiles and Prognostic Value of FABPs in Colorectal Adenocarcinomas. Biomedicines.

[22] Tian W., Zhang W., Zhang Y., Zhu T., Hua Y., Li H., Zhang Q., Xia M. (2020). FABP4 promotes invasion and metastasis of colon cancer by regulating fatty acid transport. Cancer Cell Int..

[23] Wu L., Ou G.L., Zhang W., Ma H.X., Li X.Y., Zhen Y.H., Wu H., Cao K., Li H.Y. (2025). Fatty acid-binding proteins in cancers. Int. J. Surg..

[24] Zhang Y., Zhang W., Xia M., Xie Z., An F., Zhan Q., Tian W., Zhu T. (2021). High expression of FABP4 in colorectal cancer and its clinical significance. J. Zhejiang Univ. Sci. B.

[25] Chen Y.J., Liao W.X., Huang S.Z., Yu Y.F., Wen J.Y., Chen J., Lin D.G., Wu X.Y., Jiang N., Li X. (2021). Prognostic and immunological role of CD36: A pan-cancer analysis. J. Cancer.

[26] Drury J., Rychahou P.G., He D., Jafari N., Wang C., Lee E.Y., Weiss H.L., Evers B.M., Zaytseva Y.Y. (2020). Inhibition of Fatty Acid Synthase Upregulates Expression of CD36 to Sustain Proliferation of Colorectal Cancer Cells. Front. Oncol..

[27] Drury J., Rychahou P.G., Kelson C.O., Geisen M.E., Wu Y., He D., Wang C., Lee E.Y., Evers B.M., Zaytseva Y.Y. (2022). Upregulation of CD36, a Fatty Acid Translocase, Promotes Colorectal Cancer Metastasis by Increasing MMP28 and Decreasing E-Cadherin Expression. Cancers.

[28] Fang Y., Shen Z.Y., Zhan Y.Z., Feng X.C., Chen K.L., Li Y.S., Deng H.J., Pan S.M., Wu D.H., Ding Y. (2019). CD36 inhibits β-catenin/c-myc-mediated glycolysis through ubiquitination of GPC4 to repress colorectal tumorigenesis. Nat. Commun..

[29] Gong J., Lin Y., Zhang H., Liu C., Cheng Z., Yang X., Zhang J., Xiao Y., Sang N., Qian X. (2020). Reprogramming of lipid metabolism in cancer-associated fibroblasts potentiates migration of colorectal cancer cells. Cell Death Dis..

[30] Misbah M., Kumar M., Lee K.H., Shen S.C. (2022). Identification of Novel miRNAs, Targeting Genes, Signaling Pathway, and the Small Molecule for Overcoming Oxaliplatin Resistance of Metastatic Colorectal Cancer. Biomed. Res. Int..

[31] Geng Q.S., Yang M.J., Li L.F., Shen Z.B., Wang L.H., Zheng Y.Y., Xue W.H., Zhao J. (2021). Over-Expression and Prognostic Significance of FATP5, as a New Biomarker, in Colorectal Carcinoma. Front. Mol. Biosci..

[32] Qiao C., Huang W., Chen J., Feng W., Zhang T., Wang Y., Liu D., Ji X., Xie M., Sun M. (2021). IGF1-mediated HOXA13 overexpression promotes colorectal cancer metastasis through upregulating ACLY and IGF1R. Cell Death Dis..

[33] Wen J., Min X., Shen M., Hua Q., Han Y., Zhao L., Liu L., Huang G., Liu J., Zhao X. (2019). ACLY facilitates colon cancer cell metastasis by CTNNB1. J. Exp. Clin. Cancer Res..

[34] Ha J., Daniel S., Broyles S.S., Kim K.H. (1994). Critical phosphorylation sites for acetyl-CoA carboxylase activity. J. Biol. Chem..

[35] Jiang M., Xu L., Lin W., Liu W., Zhang Y., Wang H., Zhao L. (2025). LncRNA CRCMSL interferes in phospholipid unsaturation to suppress colorectal cancer progression via reducing membrane fluidity. J. Adv. Res..

[36] Zhang C.Y., Zhang R., Zhang L., Wang Z.M., Sun H.Z., Cui Z.G., Zheng H.C. (2023). Regenerating gene 4 promotes chemoresistance of colorectal cancer by affecting lipid droplet synthesis and assembly. World J. Gastroenterol..

[37] Kelson C.O., Tessmann J.W., Geisen M.E., He D., Wang C., Gao T., Evers B.M., Zaytseva Y.Y. (2024). Upregulation of Fatty Acid Synthase Increases Activity of β-Catenin and Expression of NOTUM to Enhance Stem-like Properties of Colorectal Cancer Cells. Cells.

[38] Khiewkamrop P., Surangkul D., Srikummool M., Richert L., Pekthong D., Parhira S., Somran J., Srisawang P. (2022). Epigallocatechin gallate triggers apoptosis by suppressing de novo lipogenesis in colorectal carcinoma cells. FEBS Open Bio.

[39] Wei W., Qin B., Wen W., Zhang B., Luo H., Wang Y., Xu H., Xie X., Liu S., Jiang X. (2023). FBXW7β loss-of-function enhances FASN-mediated lipogenesis and promotes colorectal cancer growth. Signal Transduct. Target. Ther..

[40] Zaytseva Y.Y., Rychahou P.G., Gulhati P., Elliott V.A., Mustain W.C., O’Connor K., Morris A.J., Sunkara M., Weiss H.L., Lee E.Y. (2012). Inhibition of fatty acid synthase attenuates CD44-associated signaling and reduces metastasis in colorectal cancer. Cancer Res..

[41] Liao C., Li M., Li X., Li N., Zhao X., Wang X., Song Y., Quan J., Cheng C., Liu J. (2020). Trichothecin inhibits invasion and metastasis of colon carcinoma associating with SCD-1-mediated metabolite alteration. Biochim. Biophys. Acta Mol. Cell Biol. Lipids.

[42] Ran H., Zhu Y., Deng R., Zhang Q., Liu X., Feng M., Zhong J., Lin S., Tong X., Su Q. (2018). Stearoyl-CoA desaturase-1 promotes colorectal cancer metastasis in response to glucose by suppressing PTEN. J. Exp. Clin. Cancer Res..

[43] Wu T., Wan J., Qu X., Xia K., Wang F., Zhang Z., Yang M., Wu X., Gao R., Yuan X. (2023). Nodal promotes colorectal cancer survival and metastasis through regulating SCD1-mediated ferroptosis resistance. Cell Death Dis..

[44] Wang Y.N., Zeng Z.L., Lu J., Wang Y., Liu Z.X., He M.M., Zhao Q., Wang Z.X., Li T., Lu Y.X. (2018). CPT1A-mediated fatty acid oxidation promotes colorectal cancer cell metastasis by inhibiting anoikis. Oncogene.

[45] Li J., Zheng W., Wu J., Zhang J., Lv B., Li W., Liu J., Zhang X., Huang T., Luo Z. (2023). CPT1C-mediated fatty acid oxidation facilitates colorectal cancer cell proliferation and metastasis. Acta Biochim. Biophys. Sin..

[46] Li H., Chen J., Liu J., Lai Y., Huang S., Zheng L., Fan N. (2021). CPT2 downregulation triggers stemness and oxaliplatin resistance in colorectal cancer via activating the ROS/Wnt/β-catenin-induced glycolytic metabolism. Exp. Cell Res..

[47] Liu F., Li X., Yan H., Wu J., Yang Y., He J., Chen J., Jiang Z., Wu F., Jiang Z. (2022). Downregulation of CPT2 promotes proliferation and inhibits apoptosis through p53 pathway in colorectal cancer. Cell Signal.

[48] Shi B., Chen J., Guo H., Shi X., Tai Q., Chen G., Yao H., Mi X., Zhong R., Lu Y. (2025). ACOX1 activates autophagy via the ROS/mTOR pathway to suppress proliferation and migration of colorectal cancer. Sci. Rep..

[49] Zheng R., Wang Y. (2023). SLC9A5 promotes tumor growth and cell motility via ACOX1-mediated peroxisomal fatty acid oxidation. Exp. Cell Res..

[50] Wang Y., Xu C., Yang X., Liu X., Guo Z., Lin X., Li L., Huang Z. (2024). Glycerol-3-phosphate acyltransferase 3-mediated lipid droplets accumulation confers chemoresistance of colorectal cancer. MedComm.

[51] Cotte A.K., Aires V., Fredon M., Limagne E., Derangère V., Thibaudin M., Humblin E., Scagliarini A., de Barros J.P., Hillon P. (2018). Lysophosphatidylcholine acyltransferase 2-mediated lipid droplet production supports colorectal cancer chemoresistance. Nat. Commun..

[52] Katoh Y., Yaguchi T., Kubo A., Iwata T., Morii K., Kato D., Ohta S., Satomi R., Yamamoto Y., Oyamada Y. (2022). Inhibition of stearoyl-CoA desaturase 1 (SCD1) enhances the antitumor T cell response through regulating β-catenin signaling in cancer cells and ER stress in T cells and synergizes with anti-PD-1 antibody. J. Immunother. Cancer.

[53] Sugi T., Katoh Y., Ikeda T., Seta D., Iwata T., Nishio H., Sugawara M., Kato D., Katoh K., Kawana K. (2024). SCD1 inhibition enhances the effector functions of CD8(+) T cells via ACAT1-dependent reduction of esterified cholesterol. Cancer Sci..

[54] Tracz-Gaszewska Z., Sowka A., Dobrzyn P. (2023). Stearoyl-CoA desaturase 1 inhibition impairs triacylglycerol accumulation and lipid droplet formation in colorectal cancer cells. J. Cell Physiol..

[55] Zuo Q., Xu Q., Li Z., Luo D., Peng H., Duan Z. (2023). Kruppel-like factor 5 enhances proliferation, lipid droplet formation and oxaliplatin resistance in colorectal cancer by promoting fatty acid binding protein 6 transcription. Anticancer. Drugs.

[56] Zuo Q., Xu Q., Li Z., Luo D., Peng H., Duan Z. (2024). TRIM3 inhibits colorectal cancer cell migration and lipid droplet formation by promoting FABP4 degradation. Histol. Histopathol..

[57] Iftikhar R., Penrose H.M., King A.N., Samudre J.S., Collins M.E., Hartono A.B., Lee S.B., Lau F., Baddoo M., Flemington E.F. (2021). Elevated ATGL in colon cancer cells and cancer stem cells promotes metabolic and tumorigenic reprogramming reinforced by obesity. Oncogenesis.

[58] Yin H., Li W., Mo L., Deng S., Lin W., Ma C., Luo Z., Luo C., Hong H. (2021). Adipose triglyceride lipase promotes the proliferation of colorectal cancer cells via enhancing the lipolytic pathway. J. Cell Mol. Med..

[59] Sainero-Alcolado L., Garde-Lapido E., Snaebjörnsson M.T., Schoch S., Stevens I., Ruiz-Pérez M.V., Dyrager C., Pelechano V., Axelson H., Schulze A. (2024). Targeting MYC induces lipid droplet accumulation by upregulation of HILPDA in clear cell renal cell carcinoma. Proc. Natl. Acad. Sci. USA.

[60] VandeKopple M.J., Wu J., Auer E.N., Giaccia A.J., Denko N.C., Papandreou I. (2019). HILPDA Regulates Lipid Metabolism, Lipid Droplet Abundance, and Response to Microenvironmental Stress in Solid Tumors. Mol. Cancer Res..

[61] Matsubara J., Honda K., Ono M., Sekine S., Tanaka Y., Kobayashi M., Jung G., Sakuma T., Nakamori S., Sata N. (2011). Identification of adipophilin as a potential plasma biomarker for colorectal cancer using label-free quantitative mass spectrometry and protein microarray. Cancer Epidemiol. Biomark. Prev..

[62] Yang F., Li Y., Shang X., Zhu Y., Hou W., Liu Y., Hua Q., Sun Z. (2025). PLIN2 promotes colorectal cancer progression through CD36-mediated epithelial-mesenchymal transition. Cell Death Dis..

[63] Gao Y., Nan X., Shi X., Mu X., Liu B., Zhu H., Yao B., Liu X., Yang T., Hu Y. (2019). SREBP1 promotes the invasion of colorectal cancer accompanied upregulation of MMP7 expression and NF-κB pathway activation. BMC Cancer.

[64] Jin L., Zhu L.Y., Pan Y.L., Fu H.Q., Zhang J. (2021). Prothymosin α promotes colorectal carcinoma chemoresistance through inducing lipid droplet accumulation. Mitochondrion.

[65] Wang H., Chen Y., Liu Y., Li Q., Luo J., Wang L., Chen Y., Sang C., Zhang W., Ge X. (2022). The lncRNA ZFAS1 regulates lipogenesis in colorectal cancer by binding polyadenylate-binding protein 2 to stabilize SREBP1 mRNA. Mol. Ther. Nucleic Acids.

[66] Wen Y.A., Xiong X., Zaytseva Y.Y., Napier D.L., Vallee E., Li A.T., Wang C., Weiss H.L., Evers B.M., Gao T. (2018). Downregulation of SREBP inhibits tumor growth and initiation by altering cellular metabolism in colon cancer. Cell Death Dis..

[67] Dai W., Xiang W., Han L., Yuan Z., Wang R., Ma Y., Yang Y., Cai S., Xu Y., Mo S. (2022). PTPRO represses colorectal cancer tumorigenesis and progression by reprogramming fatty acid metabolism. Cancer Commun..

[68] Gou Q., Dong C., Jin J., Liu Q., Lu W., Shi J., Hou Y. (2019). PPARα agonist alleviates tumor growth and chemo-resistance associated with the inhibition of glucose metabolic pathway. Eur. J. Pharmacol..

[69] Yaghoubizadeh M., Pishkar L., Basati G. (2020). Aberrant Expression of Peroxisome Proliferator-Activated Receptors in Colorectal Cancer and Their Association with Cancer Progression and Prognosis. Gastrointest. Tumors.

[70] Mana M.D., Hussey A.M., Tzouanas C.N., Imada S., Barrera Millan Y., Bahceci D., Saiz D.R., Webb A.T., Lewis C.A., Carmeliet P. (2021). High-fat diet-activated fatty acid oxidation mediates intestinal stemness and tumorigenicity. Cell Rep..

[71] Shan J., Li X., Sun R., Yao Y., Sun Y., Kuang Q., Dai X., Sun Y. (2024). Palmitoyltransferase ZDHHC6 promotes colon tumorigenesis by targeting PPARγ-driven lipid biosynthesis via regulating lipidome metabolic reprogramming. J. Exp. Clin. Cancer Res..

[72] Zadoorian A., Du X., Yang H. (2023). Lipid droplet biogenesis and functions in health and disease. Nat. Rev. Endocrinol..

[73] Kamphorst J.J., Cross J.R., Fan J., de Stanchina E., Mathew R., White E.P., Thompson C.B., Rabinowitz J.D. (2013). Hypoxic and Ras-transformed cells support growth by scavenging unsaturated fatty acids from lysophospholipids. Proc. Natl. Acad. Sci. USA.

[74] Shihadih D., Wang X., Zushin P.H., Khodakivskyi P., Park H.M., Tso E., Shiblak J., Misic A., Louie S.M., Ward C. (2024). FATP5 Is Indispensable for the Growth of Intrahepatic Cholangiocarcinoma. Mol. Cancer Res..

[75] Yu L., Wei W., Lv J., Lu Y., Wang Z., Cai C. (2024). FABP4-mediated lipid metabolism promotes TNBC progression and breast cancer stem cell activity. Cancer Lett..

[76] Pascual G., Avgustinova A., Mejetta S., Martín M., Castellanos A., Attolini C.S., Berenguer A., Prats N., Toll A., Hueto J.A. (2017). Targeting metastasis-initiating cells through the fatty acid receptor CD36. Nature.

[77] Tao L., Mohammad M.A., Milazzo G., Moreno-Smith M., Patel T.D., Zorman B., Badachhape A., Hernandez B.E., Wolf A.B., Zeng Z. (2022). MYCN-driven fatty acid uptake is a metabolic vulnerability in neuroblastoma. Nat. Commun..

[78] Tong K., Yang C., Cai L., Yang W., Yu R., Xu J., Jiang F. (2025). Long chain fatty acid transport via SLC27A1 enhances DAG-3-P synthesis and accelerates colorectal cancer metastasis. Sci. Rep..

[79] Fhu C.W., Ali A. (2020). Fatty Acid Synthase: An Emerging Target in Cancer. Molecules.

[80] Wellen K.E., Hatzivassiliou G., Sachdeva U.M., Bui T.V., Cross J.R., Thompson C.B. (2009). ATP-citrate lyase links cellular metabolism to histone acetylation. Science.

[81] Hatzivassiliou G., Zhao F., Bauer D.E., Andreadis C., Shaw A.N., Dhanak D., Hingorani S.R., Tuveson D.A., Thompson C.B. (2005). ATP citrate lyase inhibition can suppress tumor cell growth. Cancer Cell.

[82] Gu D., Ye M., Zhu G., Bai J., Chen J., Yan L., Yu P., Lu F., Hu C., Zhong Y. (2024). Hypoxia upregulating ACSS2 enhances lipid metabolism reprogramming through HMGCS1 mediated PI3K/AKT/mTOR pathway to promote the progression of pancreatic neuroendocrine neoplasms. J. Transl. Med..

[83] Schug Z.T., Peck B., Jones D.T., Zhang Q., Grosskurth S., Alam I.S., Goodwin L.M., Smethurst E., Mason S., Blyth K. (2015). Acetyl-CoA synthetase 2 promotes acetate utilization and maintains cancer cell growth under metabolic stress. Cancer Cell.

[84] Fullerton M.D., Galic S., Marcinko K., Sikkema S., Pulinilkunnil T., Chen Z.P., O’Neill H.M., Ford R.J., Palanivel R., O’Brien M. (2013). Single phosphorylation sites in Acc1 and Acc2 regulate lipid homeostasis and the insulin-sensitizing effects of metformin. Nat. Med..

[85] Wang Y., Zhang M., Liu J., Li C., Sun N., Wu X., Wang C., Tan X., Yang Y., Qi X. (2025). Novel therapeutic strategies for targeting fatty acid oxidation in cancer. Biomark. Res..

[86] Ferdinandusse S., Denis S., van Roermund C.W.T., Preece M.A., Koster J., Ebberink M.S., Waterham H.R., Wanders R.J.A. (2018). A novel case of ACOX2 deficiency leads to recognition of a third human peroxisomal acyl-CoA oxidase. Biochim. Biophys. Acta Mol. Basis Dis..

[87] Liu X., Sun X., Mu W., Li Y., Bu W., Yang T., Zhang J., Liu R., Ren J., Zhou J. (2025). Autophagic flux-lipid droplet biogenesis cascade sustains mitochondrial fitness in colorectal cancer cells adapted to acidosis. Cell Death Discov..

[88] Rambold A.S., Cohen S., Lippincott-Schwartz J. (2015). Fatty acid trafficking in starved cells: Regulation by lipid droplet lipolysis, autophagy, and mitochondrial fusion dynamics. Dev. Cell.

[89] Deswal B., Nallanthighal S., Nikpayam E., Minhas Z., Paul A., Goo Y.H., Cheon D.J. (2025). Lipid droplet-associated hydrolase (LDAH) knockdown enhances TAG hydrolysis and promotes ovarian cancer progression and chemoresistance. Oncogenesis.

[90] Kostecka L.G., Mendez S., Li M., Khare P., Zhang C., Le A., Amend S.R., Pienta K.J. (2024). Cancer cells employ lipid droplets to survive toxic stress. Prostate.

[91] Jarc E., Kump A., Malavašič P., Eichmann T.O., Zimmermann R., Petan T. (2018). Lipid droplets induced by secreted phospholipase A(2) and unsaturated fatty acids protect breast cancer cells from nutrient and lipotoxic stress. Biochim. Biophys. Acta Mol. Cell Biol. Lipids.

[92] Wu H., Han Y., Rodriguez Sillke Y., Deng H., Siddiqui S., Treese C., Schmidt F., Friedrich M., Keye J., Wan J. (2019). Lipid droplet-dependent fatty acid metabolism controls the immune suppressive phenotype of tumor-associated macrophages. EMBO Mol. Med..

[93] Zhang Y., Yu S., Yeernuer D., Liu W., Xu Z., Feng W., Lv Z., Liu X., Tan P., Zheng M. (2025). IL33-induced lipid droplet formation in mature low-density neutrophils drives colorectal cancer liver metastasis. Cell Mol. Immunol..

[94] Shen C., Liu J., Liu H., Li G., Wang H., Tian H., Mao Y., Hua D. (2024). Timosaponin AIII induces lipid peroxidation and ferroptosis by enhancing Rab7-mediated lipophagy in colorectal cancer cells. Phytomedicine.

[95] Lee H., Horbath A., Kondiparthi L., Meena J.K., Lei G., Dasgupta S., Liu X., Zhuang L., Koppula P., Li M. (2024). Cell cycle arrest induces lipid droplet formation and confers ferroptosis resistance. Nat. Commun..

[96] Bougarne N., Weyers B., Desmet S.J., Deckers J., Ray D.W., Staels B., De Bosscher K. (2018). Molecular Actions of PPARα in Lipid Metabolism and Inflammation. Endocr. Rev..

[97] Torres J.L., Usategui-Martín R., Hernández-Cosido L., Bernardo E., Manzanedo-Bueno L., Hernández-García I., Mateos-Díaz A.M., Rozo O., Matesanz N., Salete-Granado D. (2022). PPAR-γ Gene Expression in Human Adipose Tissue Is Associated with Weight Loss After Sleeve Gastrectomy. J. Gastrointest. Surg..

[98] Chen Z., Han F., Du Y., Shi H., Zhou W. (2023). Hypoxic microenvironment in cancer: Molecular mechanisms and therapeutic interventions. Signal Transduct. Target. Ther..

[99] Dong S., Li S., Wang X., Liang S., Zhang W., Li L., Xu Q., Shi B., Cheng Z., Zhang X. (2022). CD147 Mediates 5-Fluorouracil Resistance in Colorectal Cancer by Reprogramming Glycolipid Metabolism. Front. Oncol..

[100] Zhang B., Zhu Y., Tang Y., Liu L., Liu Y., Li Y., Yu W., Lu L. (2025). The mediator subunit complex protein MED15 promotes lipid deposition and cancer progression during hypoxia. J. Biol. Chem..

[101] Zheng H., Wu Y., Guo T., Liu F., Xu Y., Cai S. (2020). Hypoxia Induces Growth Differentiation Factor 15 to Promote the Metastasis of Colorectal Cancer via PERK-eIF2α Signaling. Biomed. Res. Int..

[102] Wen Y.A., Xing X., Harris J.W., Zaytseva Y.Y., Mitov M.I., Napier D.L., Weiss H.L., Evers B.M., Gao T. (2017). Adipocytes activate mitochondrial fatty acid oxidation and autophagy to promote tumor growth in colon cancer. Cell Death Dis..

[103] Xu S., Peng X., Wang Z., Le C., Wu X., Zeng Z., Zeng S., Zhang C., Qiu M., Zou X. (2025). FABP7-mediated lipid-laden macrophages drive the formation of pre-metastatic niche and liver metastasis. Int. J. Biol. Sci..

[104] Ni T., He Z., Dai Y., Yao J., Guo Q., Wei L. (2017). Oroxylin A suppresses the development and growth of colorectal cancer through reprogram of HIF1α-modulated fatty acid metabolism. Cell Death Dis..

[105] Singhal R., Mitta S.R., Das N.K., Kerk S.A., Sajjakulnukit P., Solanki S., Andren A., Kumar R., Olive K.P., Banerjee R. (2021). HIF-2α activation potentiates oxidative cell death in colorectal cancers by increasing cellular iron. J. Clin. Investig..

[106] Huang S., Wang H., Cao J., Pang Q. (2025). Hypoxia and lipid metabolism related genes drive proliferation migration and immune infiltration mechanisms in colorectal cancer subtyping. Sci. Rep..

[107] Rolver M.G., Holland L.K.K., Ponniah M., Prasad N.S., Yao J., Schnipper J., Kramer S., Elingaard-Larsen L., Pedraz-Cuesta E., Liu B. (2023). Chronic acidosis rewires cancer cell metabolism through PPARα signaling. Int. J. Cancer.

[108] Chipurupalli S., Ganesan R., Martini G., Mele L., Reggio A., Esposito M., Kannan E., Namasivayam V., Grumati P., Desiderio V. (2022). Cancer cells adapt FAM134B/BiP mediated ER-phagy to survive hypoxic stress. Cell Death Dis..

[109] Emerling B.M., Weinberg F., Snyder C., Burgess Z., Mutlu G.M., Viollet B., Budinger G.R., Chandel N.S. (2009). Hypoxic activation of AMPK is dependent on mitochondrial ROS but independent of an increase in AMP/ATP ratio. Free Radic. Biol. Med..

[110] Kiesel V.A., Sheeley M.P., Hicks E.M., Andolino C., Donkin S.S., Wendt M.K., Hursting S.D., Teegarden D. (2022). Hypoxia-Mediated ATF4 Induction Promotes Survival in Detached Conditions in Metastatic Murine Mammary Cancer Cells. Front. Oncol..

[111] König S., Strassheimer F., Brandner N.I., Schröder J.H., Urban H., Harwart L.F., Hehlgans S., Steinbach J.P., Ronellenfitsch M.W., Luger A.L. (2024). Superoxide dismutase 1 mediates adaptation to the tumor microenvironment of glioma cells via mammalian target of rapamycin complex 1. Cell Death Discov..

[112] Wakui M., Kawai K., Mizushima T., Nishime C., Serizawa A., Suemizu H., Asakura K., Yamauchi Y., Hayashida T., Suematsu M. (2019). Fatty Acid β-Oxidation-dependent and -independent Responses and Tumor Aggressiveness Acquired Under Mild Hypoxia. Anticancer. Res..

[113] Seo J., Yun J., Fukuda J., Chun Y.S. (2021). Tumor-intrinsic FABP5 is a novel driver for colon cancer cell growth via the HIF-1 signaling pathway. Cancer Genet..

[114] Liu D., Zhu H., Cheng L., Li R., Ma X., Wang J., Wang J., Zhang S., Li Y., Shu K. (2024). Hypoxia-induced galectin-8 maintains stemness in glioma stem cells via autophagy regulation. Neuro Oncol..

[115] Saini K.K., Chaturvedi P., Sinha A., Singh M.P., Khan M.A., Verma A., Nengroo M.A., Satrusal S.R., Meena S., Singh A. (2023). Loss of PERK function promotes ferroptosis by downregulating SLC7A11 (System Xc^−^) in colorectal cancer. Redox Biol..

[116] Zheng Y.N., Lou S.Y., Lu J., Zheng F.L., Tang Y.M., Zhang E.J., Cui S.L., Zhao H.J. (2024). Selective PI3Kδ inhibitor TYM-3-98 suppresses AKT/mTOR/SREBP1-mediated lipogenesis and promotes ferroptosis in KRAS-mutant colorectal cancer. Cell Death Dis..

[117] Li B., Hou S., Tian T., Yuan S., Li A., Feng Y., Liu Z., Mi J., Cheng Z., Lv S. (2026). Fibroblast-derived POSTN promotes colorectal cancer progression under high-fat diet by reprogramming fatty acid metabolism in tumor cell. Cell Commun. Signal.

[118] Peng S., Li Y., Huang M., Tang G., Xie Y., Chen D., Hu Y., Yu T., Cai J., Yuan Z. (2022). Metabolomics reveals that CAF-derived lipids promote colorectal cancer peritoneal metastasis by enhancing membrane fluidity. Int. J. Biol. Sci..

[119] Peng S., Chen D., Cai J., Yuan Z., Huang B., Li Y., Wang H., Luo Q., Kuang Y., Liang W. (2021). Enhancing cancer-associated fibroblast fatty acid catabolism within a metabolically challenging tumor microenvironment drives colon cancer peritoneal metastasis. Mol. Oncol..

[120] Pietraszek-Gremplewicz K., Olszańska J., Domagalski M., Tymińska A., Skoniecka A., Pikuła M., Nowak D. (2025). Dedifferentiation and metabolic reprogramming of human adipocytes in the tumor niche triggered by colorectal cancer cells. Biol. Res..

[121] Cheng Y.C., Chen M.Y., Yadav V.K., Pikatan N.W., Fong I.H., Kuo K.T., Yeh C.T., Tsai J.T. (2025). Targeting FABP4/UCP2 axis to overcome cetuximab resistance in obesity-driven CRC with drug-tolerant persister cells. Transl. Oncol..

[122] Hsu W.H., LaBella K.A., Lin Y., Xu P., Lee R., Hsieh C.E., Yang L., Zhou A., Blecher J.M., Wu C.J. (2023). Oncogenic KRAS Drives Lipofibrogenesis to Promote Angiogenesis and Colon Cancer Progression. Cancer Discov..

[123] Zhang C., Wang X.Y., Zhang P., He T.C., Han J.H., Zhang R., Lin J., Fan J., Lu L., Zhu W.W. (2022). Cancer-derived exosomal HSPC111 promotes colorectal cancer liver metastasis by reprogramming lipid metabolism in cancer-associated fibroblasts. Cell Death Dis..

[124] Deng S., Wang J., Zou F., Cheng D., Chen M., Gu J., Shi J., Yang J., Xue Y., Jiang Z. (2025). Palmitic Acid Accumulation Activates Fibroblasts and Promotes Matrix Stiffness in Colorectal Cancer. Cancer Res..

[125] Liu J., Zhang W., Chen L., Wang X., Mao X., Wu Z., Shi H., Qi H., Chen L., Huang Y. (2025). VSIG4 Promotes Tumour-Associated Macrophage M2 Polarization and Immune Escape in Colorectal Cancer via Fatty Acid Oxidation Pathway. Clin. Transl. Med..

[126] Gharzeddine K., Gonzalez Prieto C., Malier M., Hennot C., Grespan R., Yamaryo-Botté Y., Botté C.Y., Thomas F., Laverriere M.H., Girard E. (2024). Metabolic reprogramming of hypoxic tumor-associated macrophages through CSF-1R targeting favors treatment efficiency in colorectal cancers. J. Immunother. Cancer.

[127] Xu B., Hou X., Wang M., Qiu L., Chen X., Deng G., Zhou D., Feng Y., Han J., Meng X. (2025). Fatty acids modulate the colorectal cancer immune microenvironment via regulating the interaction and transactivation of PPARα/δ and P53. Cell Rep..

[128] Geng S., Zhu L., Wang Y., Liu Q., Yu C., Shi S., Yu S. (2024). Co-Colorectal cancer stem cells employ the FADS1/DDA axis to evade NK cell-mediated immunosuppression after co-cultured with NK cells under hypoxia. Int. Immunopharmacol..

[129] Zhang X., Yao J., Shi H., Gao B., Zhang L. (2019). LncRNA TINCR/microRNA-107/CD36 regulates cell proliferation and apoptosis in colorectal cancer via PPAR signaling pathway based on bioinformatics analysis. Biol. Chem..

[130] Du W., Zhang J., Wang Y., Li M., Cao J., Yang B., He Q., Shao X., Ying M. (2026). Palmitic acid activates c-Myc via dual palmitoylation-dependent pathways to promote colon cancer. Cell Discov..

[131] Nakata K., Komiyama S., Sekine K., Nagai M., Okawa T., Ohashi W., Nakano K., Okamura T., Kozono T., Takemura N. (2026). Dietary Stearic Acid Accelerates Intestinal Tumorigenesis via Fatty Acid-binding Protein 5 Without Promoting Obesity. Cell Mol. Gastroenterol. Hepatol..

[132] Cioccoloni G., Bonmassar L., Pagani E., Caporali S., Fuggetta M.P., Bonmassar E., D’Atri S., Aquino A. (2015). Influence of fatty acid synthase inhibitor orlistat on the DNA repair enzyme O6-methylguanine-DNA methyltransferase in human normal or malignant cells in vitro. Int. J. Oncol..

[133] Enkhbat T., Nishi M., Yoshikawa K., Jun H., Tokunaga T., Takasu C., Kashihara H., Ishikawa D., Tominaga M., Shimada M. (2018). Epigallocatechin-3-gallate Enhances Radiation Sensitivity in Colorectal Cancer Cells Through Nrf2 Activation and Autophagy. Anticancer. Res..

[134] La X., Zhang L., Li Z., Li H., Yang Y. (2019). (-)-Epigallocatechin Gallate (EGCG) Enhances the Sensitivity of Colorectal Cancer Cells to 5-FU by Inhibiting GRP78/NF-κB/miR-155-5p/MDR1 Pathway. J. Agric. Food Chem..

[135] Banerjee M., Zaytseva Y.Y., Reusch E.M., Napier D.L., Hasani S., Rychahou P., Izumi T., Cheek D.A., Li J., Flight R.M. (2026). FASN Inhibition Enhances the Efficacy of Chemotherapy in Colorectal Cancer by Inhibiting the DNA Damage Response. Cancer Res..

[136] Falchook G., Infante J., Arkenau H.T., Patel M.R., Dean E., Borazanci E., Brenner A., Cook N., Lopez J., Pant S. (2021). First-in-human study of the safety, pharmacokinetics, and pharmacodynamics of first-in-class fatty acid synthase inhibitor TVB-2640 alone and with a taxane in advanced tumors. EClinicalMedicine.

[137] Tang Y., Tang R., Tang M., Huang P., Liao Z., Zhou J., Zhou L., Su M., Chen P., Jiang J. (2020). LncRNA DNAJC3-AS1 Regulates Fatty Acid Synthase via the EGFR Pathway to Promote the Progression of Colorectal Cancer. Front. Oncol..

[138] Zhou Y., Bollu L.R., Tozzi F., Ye X., Bhattacharya R., Gao G., Dupre E., Xia L., Lu J., Fan F. (2013). ATP citrate lyase mediates resistance of colorectal cancer cells to SN38. Mol. Cancer Ther..

[139] Zabielska J., Stelmanska E., Szrok-Jurga S., Kobiela J., Czumaj A. (2025). Lipids Metabolism Inhibition Antiproliferative Synergy with 5-Fluorouracil in Human Colorectal Cancer Model. Int. J. Mol. Sci..

[140] Liu Y., Hua W., Li Y., Xian X., Zhao Z., Liu C., Zou J., Li J., Fang X., Zhu Y. (2020). Berberine suppresses colon cancer cell proliferation by inhibiting the SCAP/SREBP-1 signaling pathway-mediated lipogenesis. Biochem. Pharmacol..

[141] Wang Y., Guo D., He J., Song L., Chen H., Zhang Z., Tan N. (2019). Inhibition of fatty acid synthesis arrests colorectal neoplasm growth and metastasis: Anti-cancer therapeutical effects of natural cyclopeptide RA-XII. Biochem. Biophys. Res. Commun..

[142] Huang Y., Wang F., Lin X., Li Q., Lu Y., Zhang J., Shen X., Tan J., Qin Z., Chen J. (2023). Nuclear VCP drives colorectal cancer progression by promoting fatty acid oxidation. Proc. Natl. Acad. Sci. USA.

[143] Wei Z., Xia J., Li J., Cai J., Shan J., Zhang C., Zhang L., Wang T., Qian C., Liu L. (2023). SIRT1 promotes glucolipid metabolic conversion to facilitate tumor development in colorectal carcinoma. Int. J. Biol. Sci..

[144] Li J., Diao B., Guo S., Huang X., Yang C., Feng Z., Yan W., Ning Q., Zheng L., Chen Y. (2017). VSIG4 inhibits proinflammatory macrophage activation by reprogramming mitochondrial pyruvate metabolism. Nat. Commun..

[145] Zhang L., Qiao X., Chen M., Li P., Wen X., Sun M., Ma X., Hou Y., Yang J. (2019). Ilexgenin A prevents early colonic carcinogenesis and reprogramed lipid metabolism through HIF1α/SREBP-1. Phytomedicine.

[146] Peng Z., Song J., Zhu W., Bao H., Hu Y., Shi Y., Cheng X., Jiang M., Fang F., Chen J. (2025). Impact of sleep deprivation on colon cancer: Unraveling the KynA-P4HA2-HIF-1α axis in tumor lipid metabolism and metastasis. Mol. Metab..

[147] Zeng K., Li W., Wang Y., Zhang Z., Zhang L., Zhang W., Xing Y., Zhou C. (2023). Inhibition of CDK1 Overcomes Oxaliplatin Resistance by Regulating ACSL4-mediated Ferroptosis in Colorectal Cancer. Adv. Sci..

[148] Liao P., Wang W., Wang W., Kryczek I., Li X., Bian Y., Sell A., Wei S., Grove S., Johnson J.K. (2022). CD8(+) T cells and fatty acids orchestrate tumor ferroptosis and immunity via ACSL4. Cancer Cell.

[149] Wang W., Green M., Choi J.E., Gijón M., Kennedy P.D., Johnson J.K., Liao P., Lang X., Kryczek I., Sell A. (2019). CD8(+) T cells regulate tumour ferroptosis during cancer immunotherapy. Nature.

[150] Li H., Sun Y., Yao Y., Ke S., Zhang N., Xiong W., Shi J., He C., Xiao X., Yu H. (2024). USP8-governed GPX4 homeostasis orchestrates ferroptosis and cancer immunotherapy. Proc. Natl. Acad. Sci. USA.

[151] Iwamoto H., Abe M., Yang Y., Cui D., Seki T., Nakamura M., Hosaka K., Lim S., Wu J., He X. (2018). Cancer Lipid Metabolism Confers Antiangiogenic Drug Resistance. Cell Metab..

[152] Huang N., Ren J., Deng X., Bao Q., Huang G., Zhi S., Li Y., Li J., Hu B., Zeng D. (2025). Endothelial F3-mediated autolysosome and lipid metabolism promote resistance to anti-VEGFA therapy in metastatic colorectal cancer. Autophagy.

[153] Chen C., Zhao S., Karnad A., Freeman J.W. (2018). The biology and role of CD44 in cancer progression: Therapeutic implications. J. Hematol. Oncol..

[154] Tang F., Zhu Y., Shen J., Yuan B., He X., Tian Y., Weng L., Sun L. (2025). CD44(+) cells enhance pro-tumor stroma in the spatial landscape of colorectal cancer leading edge. Br. J. Cancer.

[155] Yang Z., Ma R., Wu W., Shi Y., Chen Y., Luo X., Li K., Wu L., Wang B., Zhang B. (2026). Targeting HIF-1α promotes ferroptosis and boosts antitumor immunity in MSS colorectal cancer. Redox Biol..

[156] Chen C., Yang Y., Guo Y., He J., Chen Z., Qiu S., Zhang Y., Ding H., Pan J., Pan Y. (2023). CYP1B1 inhibits ferroptosis and induces anti-PD-1 resistance by degrading ACSL4 in colorectal cancer. Cell Death Dis..

[157] Conche C., Finkelmeier F., Pešić M., Nicolas A.M., Böttger T.W., Kennel K.B., Denk D., Ceteci F., Mohs K., Engel E. (2023). Combining ferroptosis induction with MDSC blockade renders primary tumours and metastases in liver sensitive to immune checkpoint blockade. Gut.

[158] Lu S., Yao Z., Cheng Q., Wu J., Jiang Y., Lin H. (2024). RAS-Selective Lethal 3-Induced Ferroptosis Promotes the Antitumor Efficiency of Anti-Programmed Cell Death Protein 1 Treatment in Colorectal Cancer. Turk. J. Gastroenterol..

[159] Cremolini C., Loupakis F., Antoniotti C., Lupi C., Sensi E., Lonardi S., Mezi S., Tomasello G., Ronzoni M., Zaniboni A. (2015). FOLFOXIRI plus bevacizumab versus FOLFIRI plus bevacizumab as first-line treatment of patients with metastatic colorectal cancer: Updated overall survival and molecular subgroup analyses of the open-label, phase 3 TRIBE study. Lancet Oncol..

[160] Antoniotti C., Rossini D., Pietrantonio F., Catteau A., Salvatore L., Lonardi S., Boquet I., Tamberi S., Marmorino F., Moretto R. (2022). Upfront FOLFOXIRI plus bevacizumab with or without atezolizumab in the treatment of patients with metastatic colorectal cancer (AtezoTRIBE): A multicentre, open-label, randomised, controlled, phase 2 trial. Lancet Oncol..

[161] Antoniotti C., Rossini D., Pietrantonio F., Salvatore L., Lonardi S., Tamberi S., Marmorino F., Moretto R., Prisciandaro M., Tamburini E. (2024). Upfront Fluorouracil, Leucovorin, Oxaliplatin, and Irinotecan Plus Bevacizumab With or Without Atezolizumab for Patients With Metastatic Colorectal Cancer: Updated and Overall Survival Results of the ATEZOTRIBE Study. J. Clin. Oncol..

[162] Atanasova V.S., de Jesus Cardona C., Hejret V., Tiefenbacher A., Mair T., Tran L., Pfneissl J., Draganić K., Binder C., Kabiljo J. (2023). Mimicking Tumor Cell Heterogeneity of Colorectal Cancer in a Patient-derived Organoid-Fibroblast Model. Cell Mol. Gastroenterol. Hepatol..

[163] Głowacka K., Ibanez S., Renoult O., Vermonden P., Giolito M.V., Özkan K., Degavre C., Aubert L., Guilbaud C., Laloux-Morris F. (2024). Acid-exposed and hypoxic cancer cells do not overlap but are interdependent for unsaturated fatty acid resources. Nat. Commun..

[164] Tobias F., Hummon A.B. (2022). Lipidomic comparison of 2D and 3D colon cancer cell culture models. J. Mass. Spectrom..

[165] Vitale S., Calapà F., Colonna F., Luongo F., Biffoni M., De Maria R., Fiori M.E. (2024). Advancements in 3D In Vitro Models for Colorectal Cancer. Adv. Sci..

[166] Ooft S.N., Weeber F., Dijkstra K.K., McLean C.M., Kaing S., van Werkhoven E., Schipper L., Hoes L., Vis D.J., van de Haar J. (2019). Patient-derived organoids can predict response to chemotherapy in metastatic colorectal cancer patients. Sci. Transl. Med..

[167] Cho E.J., Kim M., Jo D., Kim J., Oh J.H., Chung H.C., Lee S.H., Kim D., Chun S.M., Kim J. (2021). Immuno-genomic classification of colorectal cancer organoids reveals cancer cells with intrinsic immunogenic properties associated with patient survival. J. Exp. Clin. Cancer Res..

[168] Zhu Y., Cai Y., Wang Q., Guo H., Xie Q., Xiang Y., Yu S., Wu B., Qiu L. (2026). Spatially Resolved Metabolomic Profiling Reveals Progression-Associated Metabolic Reprogramming in Colorectal Liver Metastasis. Metabolites.

[169] Jin L., Huang J., Guo L., Zhang B., Li Q., Li H., Yu M., Xie P., Yu Q., Chen Z. (2023). CYP1B1 promotes colorectal cancer liver metastasis by enhancing the growth of metastatic cancer cells via a fatty acids-dependent manner. J. Gastrointest. Oncol..

[170] Nissen P., Popova N.V., Gocke A., Smit D.J., Wong G.Y.M., McKay M.J., Hugh T.J., David K., Juhl H., Voß H. (2025). Proteomic Landscape of Colorectal Cancer Derived Liver Metastasis Reveals Three Distinct Phenotypes With Specific Signaling and Enhanced Survival. Mol. Cell Proteom..

[171] Kim C.Y., Kim S.C., Shin R., Kim M.J., Jeong S.Y., Park J.W., Ku J.L. (2026). Integrative multi-omics of matched primary and liver metastatic colorectal cancer organoids identifies putative molecular targets. Transl. Oncol..

